# Iodide ion-imprinted chitosan beads for highly selective adsorption for nuclear wastewater treatment applications

**DOI:** 10.1016/j.heliyon.2024.e24735

**Published:** 2024-01-20

**Authors:** Yassmin Handulle Ismail, Kean Wang, Maryam Al Shehhi, Ali Al Hammadi

**Affiliations:** a Chemical Engineering Department, Khalifa University of Science and Technology, P.O. Box 127788 Abu Dhabi, United Arab Emirates; b Emirates Nuclear Technology Center (ENTC), Khalifa University of Science and Technology, P.O. Box 127788, Abu Dhabi, United Arab Emirates; c Singapore Technology Institute, 138683, Singapore, Singapore; d Civil Infrastructure and Environmental Engineering Department, Khalifa University of Science and Technology, P.O. Box 127788 Abu Dhabi, United Arab Emirates; e Center for Catalysis and Separation (CeCas), Khalifa University of Science and Technology, P.O. Box 127788, Abu Dhabi, United Arab Emirates

**Keywords:** Chitosan, Ion-imprinting, Iodide ion, Nuclear wastewater, Batch adsorption

## Abstract

Iodide ions from radioactive iodine isotopes are common contaminants present in nuclear wastewater from nuclear power plants which are considered hazardous contaminants to be released in water sources even at low concentrations due to their association with metabolic disorders, therefore its removal from the nuclear wastewater effluents is necessary. Chitosan beads are natural and cost-efficient adsorbents that have been used for ion removal from wastewater. However, issues of poor selectivity persist in achieving high-efficiency iodide ion removal. In this study, ion-imprinted chitosan beads (IIC) have been synthesized using the phase-inversion method, IIC beads were modified by cross-linking with epichlorohydrin (IIC-EPI) and modified by cross-linking with epichlorohydrin and silicon dioxide nanoparticles (IIC–SiO_2_-EPI). Through 4 h of batch adsorption experiments, IIC beads achieved a maximum adsorption capacity (Q_e_) of 0.65 mmol g^−1^ and showed more preference for the iodide ions compared to the non-imprinted chitosan beads which achieved a maximum adsorption capacity of 0.27 mmol g^−1^ at pH 7. While the modified beads IIC-EPI and IIC-SiO_2_-EPI beads have boosted the adsorption capacities to 0.72 mmol g^−1^ and 0.91 mmol g^−1^. Scanning electron microscopic cross-sectional images have shown more pores and cavities than the surface images which agrees with the multilayer heterogeneous diffusion suggested by the Freundlich adsorption isotherm, that the experimental data has fitted. Adsorption kinetic data have fitted the Pseudo-second-order model as well as the Weber and Morris intraparticle model, which suggest an intraparticle pore diffusion adsorption mechanism, with the involvement of the physical electrostatic interactions with the cationic chitosan surface.

## Graphical abstract

**Figure:**
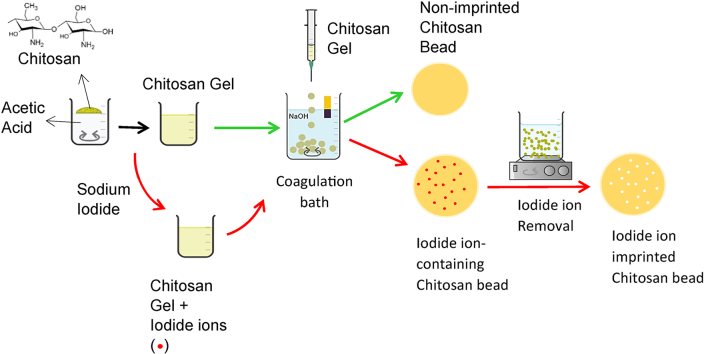

## 1 Introduction

Nuclear power plants (NPPs) are an important source of renewable energy, where metal atoms (e.g. uranium) split through nuclear fission reactions and produce the heat needed to generate electricity [1]. Besides energy, the fission reactions can produce radioactive products such as gaseous iodine isotopes that dissolve into the coolant used in the reactor and are found in liquid wastes [2]. Some of the iodine isotopes such as ^129^I have a very long half-life of 1.57 × 10^7^ years, which increases its priority to be removed [[3], [4], [5]]. During the accident of the Fukushima Daiichi nuclear plants in 2011, the leakage of iodine isotope ^131^I was reported [2], such accidents can be prevented by the proper removal of these radioactive isotopes from the NPPs aqueous waste immediately. Where long exposure to such isotopes can cause metabolic disorders and cancerous diseases [6].

The currently used methods of nuclear wastewater treatment such as filtration, evaporation, ion exchange, or chemical separation, elaborated in Table 1, have shown their applicability with heterogenous nuclear waste effluents [7]. Ion exchange adsorbents are theoretically and experimentally more convenient for the removal the radioiodine, which are commonly found completely dissolved as ions such as iodide I^−^ and iodate IO_3_^−^ in wastewater or aqueous solutions [8,9]. Chemical adsorbents have shown higher adsorption capacities than physical adsorbents but with more risk of non-reusability and the formation of secondary contaminants [10,11]. Therefore, this research aims to synthesize adsorbents that can selectively detect and adsorb iodide ions in aqueous solutions at very low concentrations.

**Table 1:** Traditional Separation procedures used for Nuclear Wastewater Treatment.

Treatment Method	Applications	Procedure	Advantages	Drawbacks	References
Evaporation	Low level radioactive effluents with high salt content and heterogenous chemical compositions.	Evaporating water leaves behind non-volatile components such as salt and radionuclides.	Very simple process with notable volume reduction results.	Possibility of corrosion, scaling, or foaming.	[7]
Chemical Precipitation	Low activity effluents with high salt and mud contents.	Separation of radionuclides by either precipitation, coagulation, or adsorption by insoluble compounds.	Relatively advanced suspended particle removal.	Requires combination with other methods due to its low decontamination results.	[7,12]
Solid-Phase Separation	Remove settled or suspended solid contents.	Removal of solid matters via separation equipment (e.g., filters, centrifuges, and hydrocyclones).	Typically, it removes particles down to submicron sizes.	Separation equipment might get exhausted and need replacement.	[7]
Ion Exchange	Efficient for waste solutions with low solids and salt concentrations, with radionuclides that are available in their ionic form.	An ion-exchange material filled fixed bed where contaminated effluent passes through from bottom to top or vice versa.	Ion exchange materials use their active sites to adsorb ions, which can be regenerated after saturation.	A semicontinuous process that requires maintenance efforts (e.g., regeneration, flushing, and refilling operations).	[7,13]

**Fig. 1:**
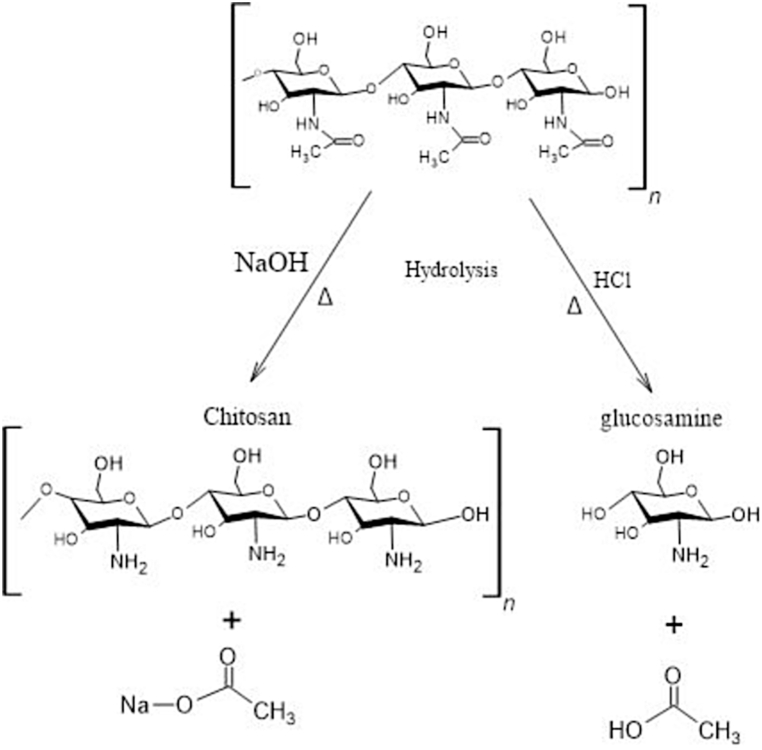
Basic (NaOH) and acidic (HCl) hydrolysis of chitin to chitosan and glucosamine, respectively.

Natural bio-adsorbents with abundant natural sources have shown their superiority in their biodegradability, availability, and ease-to-modifications [14,15]. Different biopolymeric adsorbents that are used for the removal of pollutants and ions from industrial wastewater are shown in Table 2. Due to the availability and flexibility of chitosan (CS) and its cationic nature at low pH mediums, CS has been used in this study to prepare the adsorbents. Chitosan is a naturally available polysaccharide synthesized by the deacetylation of the chitin that is found in the fungal cell walls of crustaceans, and some insects [16,17]. Chitosan is prepared by the deacetylation of Chitin. The chitin structure, shown in Fig. 1, consists of glucose units, and acetylglucosamine, which are linked through glycosidic bonds in β-(1 → 4) [17]. Chitin can produce other biopolymers through basic and acidic hydrolysis. Basic hydrolysis of chitin is more frequently used to produce chitosan, and this is due to the susceptibility of the glycosidic bonds to the acids [18]. In acid hydrolysis, a diluted acid is used to transform polysaccharides into monosaccharides at high temperatures and pressures [[19], [20], [21]]. Glucosamine is a common product of the hydrolysis of chitin with hydrochloric acid (HCl) as shown in Fig. 1. In Basic hydrolysis, treatment with an alkaline sodium hydroxide (NaOH) solution at boiling temperature permits the removal of acetylated groups and leads to the formation of Chitosan Fig. 1, which is also called the deacetylation of the chitin [22]. Other types of deacetylation involve enzymatic deacetylation, where the chitin deacetylase enzymes (EC 3.5.1.41) catalyze the hydrolysis reaction of the acetamido groups in the N-acetylglucosamine units in the chitin through a series of multiple attacks on the acetylglucosamine monomer units, which removes the acetic groups and generates glucosamine units that contain primary amino groups (-NH_2_) [18].

**Table 2:** Summary of the Biopolymeric Adsorbents used for Wastewater Treatment.

Biopolymer	Advantages	Drawbacks	References
Chitosan	1. Second most abundant polysaccharide. 2. Flexible to physical and chemical modifications. 3. Active sites of hydroxyl and primary amino functional groups. 4. pH responsiveness and stability in acidic environments. 5. Inherent biocompatibility.	1. High humidity sensitivity. 2. Cationic nature in low pH, causing low performance for cations adsorption.	[23,24]
Alginate/Sodium alginate	1. Water soluble. 2. Biocompatible and moderate gelation conditions. 3. Binds with multivalent cations.	1. High rigidity and fragile. 2. Low in elasticity and mechanical properties. 3. Lacks primary amino groups that can protonate at lower pH conditions.	[25]
Cellulose	1. First most abundant polysaccharide. 2. Facile to chemical modifications. 3. Good mechanical properties 4. Safely disposable	1. Lacks primary amino groups that can protonate at lower pH conditions.	[25]
Gelatin	1. Water-soluble polymer 2. Amphoteric nature 3. Active sites for electrostatic interactions of hydroxyl, carboxyl, and amino functional groups.	1. Low reported adsorption capacities. 2. Difficulty of adsorbate ions separation or regeneration. 3. Poor mechanical stability.	[25,26]
Agarose	1. Hydrophobic and biocompatible. 2. Nontoxic and biodegradable. 3. Great swelling capacity. 4. Chemically stable and neutrally charged. 5. Requires mild conditions of gelation.	1. Lack of amino functional groups that can protonate at lower pH conditions. 2. Relatively low stability in acidic environments. 3. Slow degradation rates.	[25,27,28]

Due to the hydroxyl groups and the amino groups, chitosan has reported a stronger affinity to numerous compounds compared to Chitin. It also has shown more flexibility in chemical and physical modifications, which justifies its various applications [23]. Chitosan has been synthesized in many forms for different applications, such as films [29], nanofibers [30], and beads [14]. CS spherical beads, unlike the other forms, can perform as good packing materials for adsorption units with convenient surface area and porosity. Various procedures have been reported for the chitosan beads production, Table 3 compares different preparation procedures for chitosan beads, among them the phase inversion synthetic method have reported the richest in polymer, the fastest, and the least disruptive procedure to the chitosan's structure.

**Table 3:** Synthetic methods of chitosan beads.

Synthetic Method	Description	Strengths	Limitations	References
Bulk Polymerization	Immersing the chitosan-containing gel into a water bath at 40 °C for 3 h, where the resulting products are ground and pressed through a mesh sieve to produce granules.	1) Simple 2) Inexpensive	1) Preparation of the reactants might be time-consuming. 2) Reduction in the yielded polymer due to grinding. Grinding might change in the polymer's shape which influences its affinity and binding site ability	[31,32]
Suspension Polymerization	Chitosan monomers are suspended in a continuous-phase solution, usually water, stabilizers or surfactants can also be added to the solution to act as microreactors. Each monomer droplet starts to polymerize as a miniature version of the bulk polymerization process, and they end up transforming gradually and completely at the end into polymeric particles.	1) More homogenous and well-defined binding sites compared to bulk polymerization. 2) No grinding in the procedure.	1) Time-consuming 2) Formed beads are not rich with only the polymer.	[33,34]
Phase Inversion Method	A homogeneous polymer solution is dropped or immersed into a continuously rotating coagulation bath.	1) Rapid procedure 2) Immediate beads formation 3) Polymer-rich beads with homogenous structure 4) No disruption to the polymer's structure.	1) Uneven surface pores distribution.	[35,36]

**Fig. 2:**
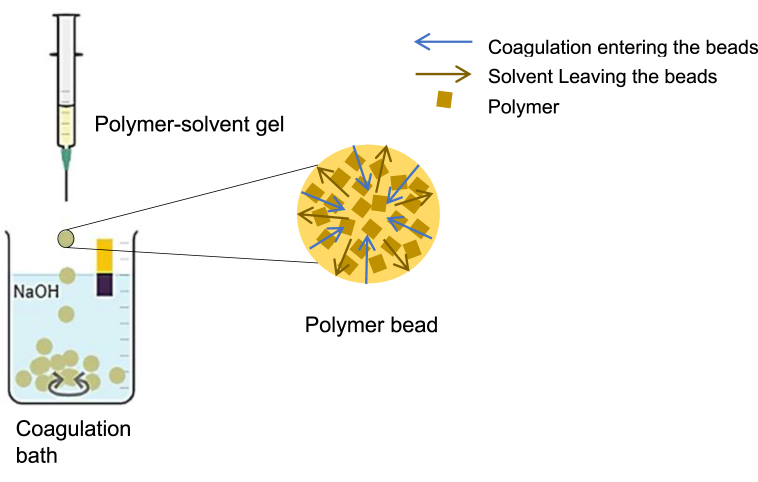
Schematic representation of phase inversion method for composite beads of polymer (chitosan-solvent (acetic acid) gel and non-solvent coagulation bath of Sodium hydroxide (NaOH).

Phase inversion is a process where a homogeneous polymer solution is dropped or immersed into a coagulation bath that is water in many applications as shown in Fig. 2. The coagulation solution de-mixes the polymer into two phases, a polymer-rich phase, and a liquid-rich phase [36]. This method is also called immersion precipitation, and it's a common membrane formation technique, however, if the coagulation bath used is in a continuous rotation while the polymer solution is dropped into it, then polymer spherical beads are more likely to be formed instead of membranes. In this method, each polymer, the solution used to dissolve the polymer, and the nonsolvent used as a coagulation bath are important factors for its success. The increase in the polymer concentration has been reported to influence the porosity of the formed beads, which reduces its pore size [35,36]. The choice of the solvent and the nonsolvent solutions is critical to the morphology and performance of the polymer beads. The more soluble the polymer is in the solvent, the more porous the resulting beads are. Once the polymer is dissolved in the solution, the polymer solvent must be capable of penetrating the nonsolvent in the coagulation bath and the nonsolvent can also be capable of penetrating the polymer solution. In addition to these factors, precipitation time, and temperature of the solution have also reported their effect on the beads formed. Various polymeric adsorbents are fabricated in the form of beads or granules due to their applicability in packed bed columns [13].

Due to the presence of the primary amino groups in the chitosan's structure, chitosan is a strong base, and when it's in contact with an acidic solution, the amino groups get protonated and become a polyelectrolyte and dissolve completely in acidic solutions [37], the CS prepared beads would possibly dissolve in acidic mediums, this can be avoided by cross-linking the CS chains. Cross-linking is one of the most effective chitosan modifications, as it allows for chitosan stabilization in an acidic medium (pH < 7). Chemical cross-linking usually involves the use of a cross-linking agent that can react with the chitosan's functional groups and form linkages among the chitosan chains. Cross-linkers such as diepoxyoctane [38], Penta sodium tripolyphosphate [39], glutaraldehyde [40,41], and epichlorohydrin (EPI) [42] have been used to modify the chitosan's structures. A comparison among previously experimented chitosan cross-linkers is found in Table 4, which shows that the EPI is the better cross-linking agent of chitosan's chains, and this is due to the chemical reactions occurring between the –OH groups of the CS molecules and the EPI molecule, and preserving the unreacted –NH_2_ groups that can perform excellent adsorption sites for anions by attraction as it protonates at lower pH conditions [43]. Other modifications involve inorganic nanoparticles to the CS's structure. Different studies have modified CS by grafting it with nanoparticles, as more details of these experiments can be found in Table 5. SiO_2_ nanoparticles have reported lower toxicity, higher porosity, adsorption capacity, scalable synthetic availability, and cost-efficient modifier to the chitosan's beads, and therefore it was used to modify the CS beads in this study [[44], [45], [46]].

**Table 4:** Recent applications of modified CS by cross-linking for the removal of adsorbates from aqueous solutions.

Cross linker	Adsorbate	Chitosan Modifications	References
Diepoxyoctane	Cr(VI)	1. An increase in chitosan functional groups got involved in cross-linking reactions with increasing the cross-linkers concentration. 2. Increase in the adsorbate uptake and performance with the increase of the cross-linking temperature and time to a certain limit to avoid occupying adsorption sites.	[38]
Penta sodium tripolyphosphate	Dye	1. Cross-linker agents accelerated the sorption process.	[39]
Glutaraldehyde	Various pollutants (proteins, fluoride, dyes, etc.).	1. Cross-linked chitosan beads have shown a decrease in crystallinity. 2. Cross-linked beads have shown less regular structure, due to the breakage of intra-molecular and inter-molecular hydrogen bonds of native beads. 3. Smoother surfaces with more porous cross-sectioned microspheres.	[40,41]
	Cd(II) ion	1. An increase in the cross-linked polymer space among its chains and surface area eases binding to the active sites.	[47]
Epichlorohydrin	N/A	1. Cross-linked microspheres have harder, smoother, and less porous surfaces. 2. Matrix compaction was observed due to cross-linking.	[41]
	Cr (VI)	1. Non-cross-linked chitosan beads show more adsorption performance stability in different pH compared to cross-linked chitosan beads. 2. Cross-linked beads improved the adsorption capacity of adsorbate in contact time of 30 min and pH = 3 compared to native chitosan beads.	[42]

**Table 5:** Recent applications of modified CS adsorbents via grafting for the removal of contaminants from aqueous solutions.

Nanoparticle	Adsorbate	Chitosan Modifications	References
TiO_2_	Anionic Dyes N/A	1) Chemically stable, high surface area, nontoxic, enhanced adsorption performance. 2) Monolayer adsorption pattern and homogeneous distribution of the active sites. 1) Structurally defined and standalone nature of pores (not interconnected). 2) Smaller pores than native chitosan adsorbents but numerous and large enough.	[[48], [49], [50]]
ZnO	N/A Anionic Dyes	1) Enhanced tensile strength. 1) Zn nanoparticles could restrict the movability of the chitosan chains. 2) Smooth and tight surface that increases with ZnO content due to interfacial interaction between ZnO nanoparticles and chitosan. 3) More thermal stability compared to native CS beads. 4) CS/ZnO nanocomposites reported a decomposition temperature of 275 °C while native CS is 230 °C.	[[51], [52], [53]]
FeO	Cr(VI) anions Naphthalene	1) Fe–O functional groups are involved in Cr(VI) anions adsorption through electrostatic interactions. 2) Homogeneous monolayer adsorption due to its fitting to Langmuir models. 1) Low cost, low toxicity, large surface area, and biodegradability. 2) Easy removal of adsorbent for further reuse. 3) Enhanced stability in acidic and oxidative mediums due to the immobility of FeO nanoparticles in the chitosan structure. 4) Suitable for the adsorption of adsorbates that are responsive to magnetic fields in which the FeO changes the magnetization dynamics of the beads.	[48,54]
SiO_2_	Anionic dye (AR73) N/A	1)Enhanced Adsorption capacity of the dye anions. 1) Improve the mechanical, interfacial, and thermal properties. 2) Increased surface area of the adsorbents especially with the smaller size SiO_2_ nanoparticles. 3) Improved surface porosity and adsorption capacity. 4) Most abundant components in the earth's crust. 5) Low toxicity, excellent chemical stability, scalable synthetic availability, and low cost. 6) Adjustable pore structure 7) Availability of silanol groups with the ease of modification.	[55] [[44], [45], [46]] [56]

Nuclear wastewater can be produced during the fabrication of nuclear fuel and spent fuel reprocessing has been found highly acidic as the reproduction processes involve using nitric acid [[57], [58], [59]]. Therefore, nuclear wastewater pH can have few fluctuations depending on the processes involved. Fission products contain radioactive isotopes and their corresponding ions. The ionic strength of the radioactive cations and anions in nuclear wastewater can significantly affect the purification process used for wastewater treatment such as adsorption. Salts (e.g. NaCl), and ions such as NO_3_^−^, SO_4_^2−^, ^137^Cs^+^, and ^90^Sr^2+^, are all considered sources of ionic competitiveness on the adsorption sites of ion-exchange-based nuclear wastewater purification methods [[60], [61], [62], [63]].

Recently, adsorbents such as bentonite clay [64], chitosan microspheres [5], and metal-organic frameworks [65] have been synthesized and achieved high adsorption capacities of their targeted adsorbates, but due to the lack of the exclusivity of the pores of these adsorbents, the co-existence of other molecules have reduced the achieved adsorption capacity notably. Regardless of the modification made to the chitosan adsorbents, selectivity is still an issue. The novelty of this work lies in preparing highly selective beads that can detect iodide ions at very low concentrations by using the molecular imprinting technique of the polymer producing molecularly imprinted polymers (MIP). MIPs are polymers that are synthesized to perfectly match the templates of a specific compound based on its spatial structure, where the molecule desired to be adsorbed (adsorbate) binds to binding sites and cavities on the MIP surface [66]. The MIPs technique has been inspired by the biological antibody-antigen systems. Where the adsorbate and the adsorbent selectively bind with each other achieving the “lock and key” mechanism [67].

**Fig. 3:**
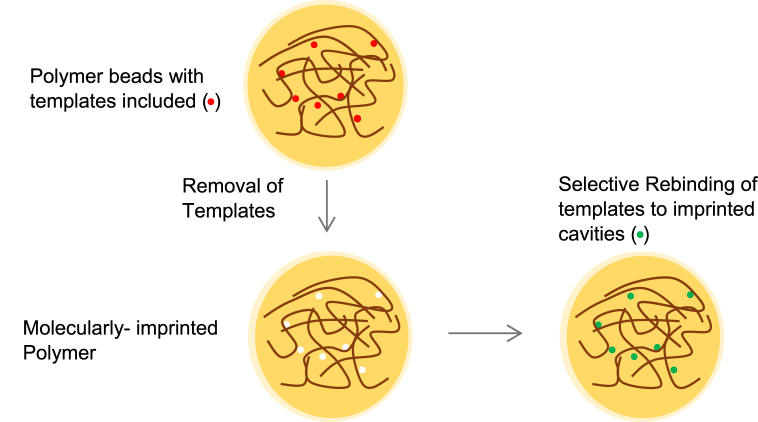
Schematic representation of the molecular imprinting process.

MIPs achieve selectivity, specificity, and stability that may not be found all at once in conventional adsorbents. The imprinting process is briefly illustrated in Fig. 3. The monomers or the polymers of the MIP polymerize around the template molecule, where the template may or may not interact with these monomers/polymers, and a cavity in the monomer/polymer forms. Later, the template molecules are removed, leaving a molecularly imprinted cavity, which is specific to adsorb the template molecules. This polymer with the selectively made cavity is ready to adsorb or bind with the molecule of interest (adsorbate) [67]. The cavities made in the polymer matrix will have a memory of the shape, size, and chemical functionality that highly corresponds to the template molecule, which allows the polymer to rebind easily and immediately to template molecules or those that are closely related to them. In the process shown in Fig. 3 the functional monomers and cross-linkers had been polymerized in the template molecule, which is also called copolymerization [68].

Chitosan beads have achieved notable results in the removal of contaminants from wastewater such as Ag^+^ ions [69], F^−^ ions [70], and I^−^ ions [2,71]. None have achieved the selective removal of the ions with the co-existence of other ions, which can be solved through the molecular imprinting technique, which is what the study targets. Recent studies have shown the successful selective removal of metal ions from aqueous solutions using ion-imprinted chitosan adsorbents such as Cd^2+^ ions [72], Pb^2+^ ions [73], and Hg^2+^ ions [74], which all have achieved higher selectivity in the presence of other ions with similar valence or ionic radius that could cause competitiveness on the ion-imprinted cavities of the adsorbents. Generally, MIPs preparation process has been reported to be complicated and in many cases time-consuming, in addition to producing particles with fewer recognition sites inside their matrices, therefore, the structure of the molecule imprinted layer is difficult to control, which might influence the adsorption capacity [75,76]. This study brings a cost-efficient and highly selective solution to remove iodide ions from nuclear wastewater.

This study involves the characterization and adsorption studies of the synthesized iodide-imprinted chitosan beads and interpretations of the synthesized beads through the adsorption patterns and mechanisms, in addition to the effect of different adsorbent, adsorbate, and operating conditions on the achieved adsorption capacity. Aspects such as the adsorption kinetics, isotherms, and the reusability of the beads are significant to understanding the prepared iodide-imprinted beads’ adsorption mechanisms and performance.

## 2 Experimental procedure

### 2.1 Materials

Medium molecular weight chitosan (CS) powder (Deacetylated chitin, poly (d-glucosamine)) from Sigma Aldrich, United States. Chemicals Epichlorohydrin (EPI), Acetic acid, Sodium Hydroxide (NaOH), Sodium Iodide (NaI), and Hydrochloric acid (HCl) are from Sigma Aldrich, United States, and were used without further purification. Silicon Dioxide nanoparticles (SiO_2_) were prepared by the Center for Membranes and Advanced Water Technology (CMAT, Khalifa University).

### 2.2 Preparation of chitosan beads

**Fig. 4:**
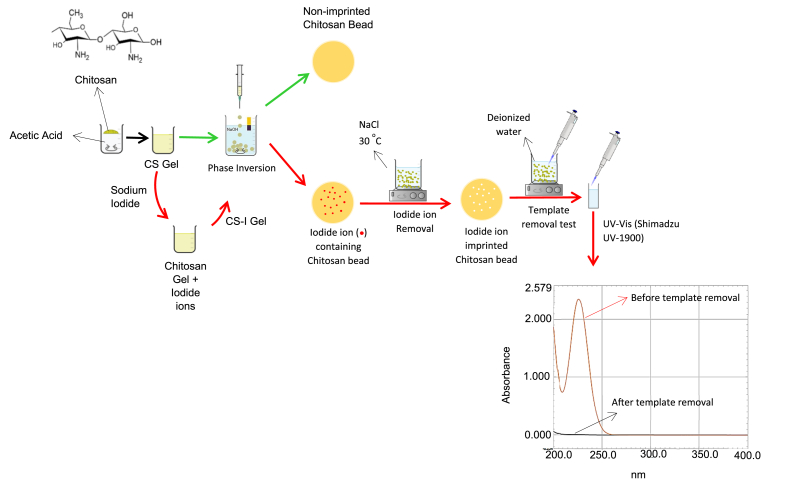
Experimental procedure for the preparation of the non-imprinted CS beads (red arrow) and ion-imprinted CS beads (green arrow). UV–vis spectra of the deionized water used to wash IIC post template removal for 48 h (horizontal line) compared to the deionized water used to wash IIC before template removal (showing a peak at 226 nm for I-), the graph is generated by 1900i (Shimadzu) software. (For interpretation of the references to color in this figure legend, the reader is referred to the Web version of this article.)

Native CS hydrogel beads (NCS) were prepared with the use of the phase inversion method, where liquid CS gel is converted to solid hydrogels. First, to prepare the CS gel, 100 mL 2 % (v/v) of diluted acetic acid with deionized water was prepared, and 2 g of CS powder was added to the diluted acetic acid and left for stirring at 100 rpm speed until a homogenous gel was formed. A 0.5 Μ NaOH solution was prepared and left at a 300 rpm speed of stirring as a coagulation bath while the CS gel, dropwise, was pumped through a syringe pump in 2.5 mL h^−1^ range into the stirring NaOH solution. A distance of 10 cm was considered between the syringe and the NaOH solution for around 1 and 30 min. The spherical hydrogel beads were formed, then filtered and rinsed repeatedly with deionized water until a neutral pH was reached, and finally with ethanol. CS hydrogel beads were stored in ethanol. The steps of the procedure are shown in detail in Fig. 4.

### 2.3 Preparation of iodide-imprinted chitosan beads

Iodide-imprinted chitosan hydrogel beads were first prepared by the addition of 2 g of CS powder into a 100 mL of 2 % (v/v) acetic acid solution and stirred for 30 min, then iodide ions templates containing powder of 1 g NaI was added to the solution shown in Fig. 4, the solution was kept for stirring at a speed of 100 rpm for two days for the iodide ion containing gel formation (CS–I). The iodide ion-containing beads were prepared in the same procedure used to prepare non-imprinted native CS beads, as the removal of the template ions should be considered later.

Solvent desorption using an ionic eluent solution was chosen to extract the I^−^ templates from the prepared beads. First, the CS beads were washed with deionized water and then added to 0.2 M of sodium chloride (NaCl) aqueous solution. NaCl is a strong electrolyte and can dissociate as ions in aqueous solutions, which allows the escape of the charged ions from the imprinted cavities via instant electrostatic attraction with the charged ions surrounding the adsorbents, but without the formation of ionic bonds due to the low concentration of ions in the aqueous solution. The beads were shaken in a NaCl solution at a speed of 150 rpm for two-three days with constant heating at 30 °C. Mild heat, as previous experimental work [77,78] has shown an acceleration in the desorption of templates while taking into account the degradation temperatures of the polymer, as increasing the heat increases the eluent dissociated ions mobility and its electrostatic interactions with ion templates in the pores. Then, ion-imprinted beads were washed with deionized water for two days and tested for the detection of I^−^ ions in the washing deionized water by the Ultraviolet–visible (UV–vis) spectra measured using UV–vis spectrometer UV-1900i (Shimadzu) as shown in Fig. 4. If the I^−^ ions were detected in the washing de-ionized water, this proves that the I^−^ ions are still in the CS matrix as they can escape to the surrounding solution.

For regenerating the binding sites of ion-imprinted CS beads the beads were stirred with a 0.5 M solution of sodium hydroxide (NaOH), where positive Na^+^ ions in the aqueous solution can electrostatically induce the escape of any possible Cl^−^ ions in the cavities, and to restore the CS beads to its original state, since NaOH was used as a coagulation solution of the beads and it is also a good eluent solution when it comes to dealing with charged ions as reported in Ref. [79]. Then, the beads were washed with deionized water until a neutral pH, followed by ethanol washing and storing them in ethanol as wet beads.

### 2.4 Preparation of modified iodide-imprinted chitosan beads

For cross-linking CS beads with EPI, the EPI/IIC (w/w) weight ratios (0.2, 0.4, 0.6, 0.8, and 1) were studied for this investigation. To prepare dry IIC-EPI beads, wet IIC beads were added to 50 mL of 1 M NaOH aqueous solution in a flask. Corresponding amounts of IIC beads and EPI were added according to Table 6 into 1 M NaOH solution since the EPI interacts with the chitosan chains at basic conditions. To achieve a different cross-linking ratio, Table 6 since the EPI is liquid, the equivalent volume of EPI was added to achieve the targeted weight ratio in Table 6 based on the general density equation **(1)**.

(1) $$V=\frac{m}{\rho }$$

**Fig. 5:**
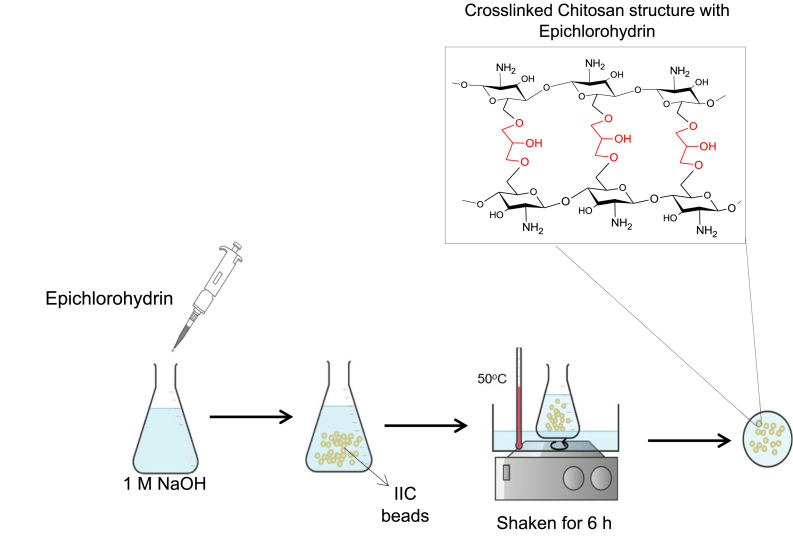
Cross-linking CS beads with EPI experimental procedure illustration.

Where ρ is the density of the crosslinker in (mL.g^−1^), m is the mass of the crosslinker in (g), and V is the volume of the crosslinker (mL). The NaOH solution with the CS beads was shaken for 6 h at 50 °C in a water bath as shown in Fig. 5. IIC-EPI beads are washed with de-ionized water, then with ethanol, and kept in ethanol for storage.

**Table 6:** Cross-linking conditions of Wet CS Beads with EPI.

Crosslinking ratio (wt %)	Equivalent Dry CS bead weight (g)	NaOH solution (mL)	Crosslinker Density (mL.g^−1^) [80]	Volume of crosslinker added (mL)
0.2	0.065	50	1.18	0.0110
0.4		50		0.0220
0.6		50		0.0330
0.8		50		0.0441
1.0		50		0.0551

The second modification made to the beads is the modification with Silicon dioxide (SiO_2_) nanoparticles. The additional step in these samples is the addition of the SiO_2_ nanoparticles to the chitosan gel formation stage. SiO_2_ nanoparticles were first prepared by sonicating it in 50 mL of deionized water in a sonication bath for 1 h, where the deionized water started to dis-color, then the mixture was centrifuged for a few minutes to separate the SiO_2_ nanoparticles from the deionized water. The residual SiO_2_ was added to the CS gel and left for stirring until the nanoparticles were completely dissolved in the iodide ion-containing CS-I-SiO_2_ gel. Two (g SiO_2_:g CS) weight ratios were tested; (0.5:1) and (0.2:1).

### 2.5 Measurement of the equivalent dry weight of wet beads

Wet chitosan beads have been used in this study for adsorption experiments. However, the weight of a dosage of wet beads using a digital balance will increase the error since the wet samples contain water to some extent. Therefore, the equivalent weight of 100 dry beads of each of the samples has been measured and used to estimate the average weight for each 1 dry bead and assume it was the weight of 1 wet bead. This information can help count the number of individual wet beads needed to reach a target adsorbent dosage. For example, the weight of 100 dry beads of NCS was measured to be 53 mg, meaning each dry NCS bead weighs 0.53 mg, thus an adsorbent dosage of 113 wet NCS individual beads, will be equivalent to 60 mg of dry NCS beads, the same counting method was used in determining the dry weight of all the study's samples as shown in Table 7.

**Table 7:** Dry and wet weight of the prepared chitosan beads.

Sample	Weight of dry 100 beads (mg)	Weight of Dry 1 bead (mg)	Number of wet beads for an x mg target dosage
Native CS beads	53	0.53	1.89x
IIC beads	54	0.54	1.85x
IIC-EPI beads	49	0.49	2.04x
IIC-SiO_2_-EPI beads	67	0.67	1.49x

### 2.6 Batch adsorption

Batch Adsorption experiments were conducted starting with iodide ion aqueous solutions prepared from NaI. All the batch adsorption parameters for all of the experiments conducted in this study are tabulated in detail in Table 8. The equilibrium concentration of the solution after adsorption, equilibrium concentration, was measured using a UV–vis spectrometer UV-1900i (Shimadzu).

**Table 8:** Operating conditions of Batch Adsorption Experiments.

Experiment	pH	Temperature [^o^C]	Ion-imprinted beads Dosagea) [mg]	NCS Dosage [mg]	Speed of Stirring [rpm]	C_0_ [mmol.L^−1^]	Duration [h]	Volume [mL]
Cross-linking Ratio	7	Room Temperature	60	–	300	2	4	50
Grafting ratio	7		60	–	300	0.05–2	4	
Adsorbent Dosage	7		10–125	10–125	300	1	4	
Speed of Stirring	7		60	60	80–700	1	1	
Solution pH	3–11		60	60	300	1	4	
Co-existing ions	7		60	60	300	1	4	
Adsorption Kinetics	7 and 5		60	60	300	1	0–4	
Adsorption Isotherms	7 and 5		60	60	300	0.05–2	4	
Recyclability	7		60	60	300	2	4	

a) Including IIC, IIC-EPI, and IIC -SiO_2_- EPI.

### 2.7 Effect of Co-existing ions on I^−^ adsorption capacity

To investigate the effect of the ionic strength on the iodide ion adsorption, the adsorption capacity was measured at different concentrations of salt (i.e. KCl) and co-existing ions such as NO_3_^−^ through the preparations of diluted solution of KCl (1 mΜ and 0.5 mΜ) and AgNO_3_ (1 mΜ and 0.5 mΜ) that were added to an iodide solution of concentration (1 mΜ) that is going through the batch adsorption experiments.

### 2.8 Solution pH effect on I^−^ adsorption capacity

The pH of the adsorbate solution is a critical factor for the adsorption capacity of a chitosan-based adsorbent. Especially when the adsorbates are anionic solutions, where their adsorption capacity depends substantially on the adsorbents’ surface charge. Experimentally, the effect of the pH of the adsorbates was adjusted by adding drops of 0.1 M HCl and 1 M NaOH solutions to the iodide solutions before batch adsorption.

## 3 Results and discussion

Non-imprinted native CS beads (NCS), iodide-imprinted CS beads (IIC), iodide-imprinted CS cross-linked with EPI beads (IIC-EPI), and iodide-imprinted CS beads cross-linked with EPI and grafted with silicon dioxide nanoparticles (IIC–SiO_2_-EPI) were all prepared and characterized in this study. For characterization, wet CS beads were freeze-dried overnight at −80 °C using a freeze dryer (Labconco Freezone), while for batch adsorption experiments, wet CS hydrogel beads were used as adsorbent without drying.

### 3.1 Morphological characterization

#### 3.1.1 Scanning electron microscope (SEM)

**Fig. 6:**
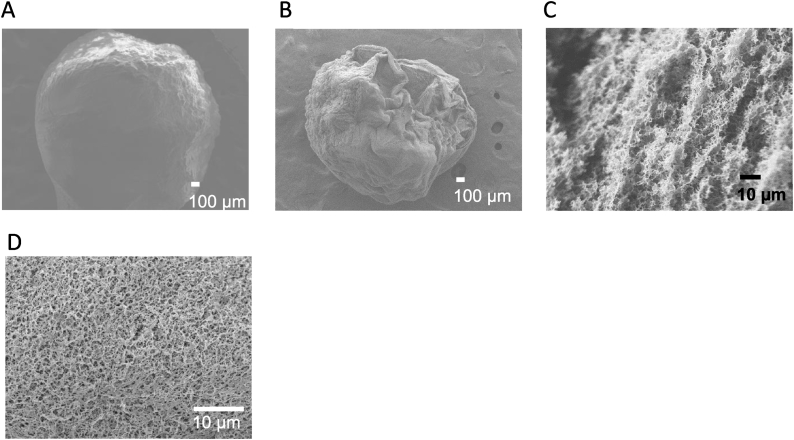
SEM images of the surface of A) NCS bead ( × 40), B) Ethanol-washed NCS bead ( × 40), C) Cross-sectional image of NCS bead ( × 1000) D) Cross-sectional image of Ethanol-washed NCS bead ( × 1400).

As wet CS beads were used in this study, the storage solution was a significant factor to consider. De-ionized water and ethanol can be used for the wet CS beads storage due to their neutral state. In addition, ethanol can remove any excess water from the pore volume, completely or partially [81]. SEM images were taken to confirm the effect of the storage solution on the beads’ porosity after freeze-drying. SEM images were taken using JEOL JSM-7610F (JEOL). Freeze-dried ethanol-stored NCS beads had a shrunk structure and irregular shape in Fig. 6B while the freeze-dried de-ionized water-stored beads with ethanol showed a more regular, smooth, and spherical structure in Fig. 6A. Ethanol-stored NCS beads in Fig. 6D have a more porous cross-sectional surface compared to the de-ionized water-stored beads in Fig. 6C, and since porosity is a good indicator of an adsorbent, ethanol was chosen to be the storage solution for the wet CS beads.

**Fig. 7:**
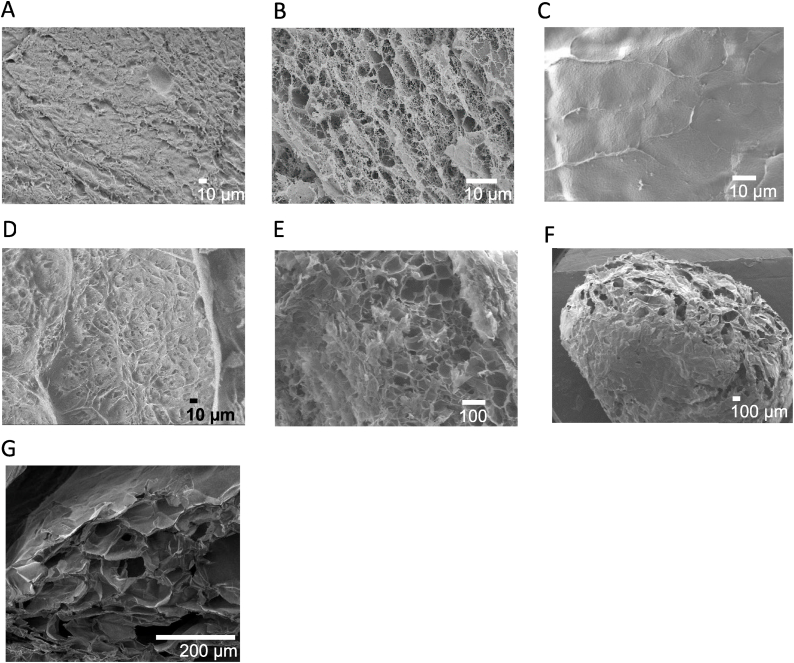
SEM images of the surface of A) Surface image of IIC bead ( × 400). B) Cross-sectional image of IIC bead ( × 1400). C) Surface image of NCS bead ( × 1300). D) Surface image of IIC-EPI bead ( × 3700). E) Cross-sectional image of IIC-EPI ( × 110). F) Surface image of IIC-SiO_2_-EPI bead ( × 40), and G) Cross-sectional image of IIC-SiO_2_-EPI ( × 500).

SEM images have also shown the effect of the CS ion imprinting on the surface morphology by comparing it to the NCS beads' surface and cross-sectional SEM images. In the case of the IIC beads, the surface image in Fig. 7A, has shown a smoother surface compared to the cross-sectional image in Fig. 7B. A similar pattern was observed with the NCS beads' surface image in Fig. 7C and cross-sectional image Fig. 6D, which indicates the multi-layer porous structure of the beads. In terms of the ion-imprinting effect, the IIC beads have shown a more heterogeneous surface structure than the NCS, in addition to the appearance of recognizable cavities/pores that appeared in the cross-sectional images of the IIC beads in Fig. 7B. The modified beads IIC-EPI in Fig. 7D and E, didn't show a major change in the morphology of the pores range due to the nature of this modification, which is a chemical modification. However, in the IIC-SiO_2_-EPI beads, the grafting with nanoparticles has caused the appearance of new macropores on the surface as shown in Fig. 7F and G., and since most of the pores are inside few layers of the beads, as observed in all the samples, these macropores can help in the exposure of the inner pores.

#### 3.1.2 Energy dispersive spectroscopy (EDS)

Energy dispersive spectroscopy (EDS) is an analytical analysis that characterizes the elemental composition of a sample's surface. EDS data were collected using JEOL JSM-7610F (JEOL). The elemental analysis can help understand the loss and emergence of elements in the synthesized samples by comparing them with a reference sample. Medium molecular weight commercial CS powder (Sigma Aldrich) was used to prepare each of the four samples of CS beads used in this study, therefore, this will be the reference for this analysis. Table 9 shows the EDS analysis of the samples. In addition to the carbon and oxygen of the CS itself, the samples have shown traces (<1 wt %) of calcium, copper, chlorine, and zinc, which mostly remains from the different process of the deacetylation of chitin and the original chitin extraction and purification processes [82]. EDS might not indicate the atomic content of carbon accurately, since the carbon tape used for the sample placed in the SEM contains mainly carbon and traces of silicon and aluminum.

**Table 9:** Elemental analysis via eds-sem of the pure CS and the synthesized beads.

Sample	Element (Atomic %)									
	C	O	Ca	Cu	Zn	Nb	Na	I	Cl	Si
Pure CS	60.62	38.50	0.10	0.35	0.23	0.20	–	–	–	–
NCS beads	71.53	28.47	–	–	–	–	–	–	–	–
IIC beads	70.47	29.53	–	–	–	–	–	–	–	–
IIC-EPI beads	61.35	38.32	–	0.12	0.07	–	–	–	0.13	–
IIC-SiO2-EPI beads	41.64	49.58	–	–	–	–	0.47	–	–	8.31

#### 3.1.3 X-ray diffraction (XRD) analysis

**Fig. 8:**
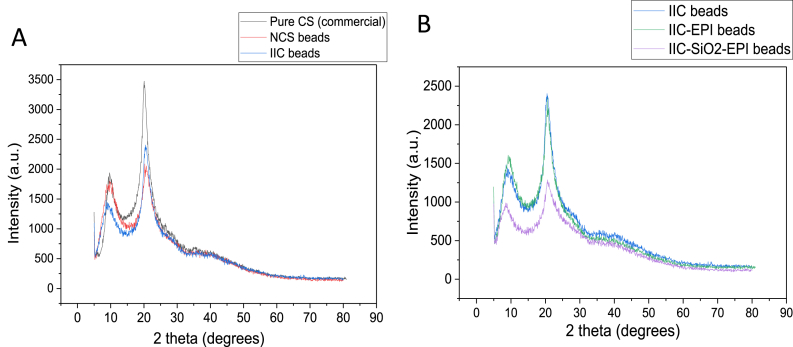
X-ray Diffractogram patterns of A) commercial CS powder, and the synthesized beads of NCS, and IIC. B) IIC, IIC-EPI, and IIC-SiO_2_-EPI beads.

X-ray diffractograms were conducted using D2 Phaser XRD under the following conditions: 30 kV, 10 mA, using a Cu tube with 1.54184 ^o^A. The corresponding intensity for each of the samples was recorded in a scattering of (2θ) from 5° to 80°. In Fig. 8A the XRD scattering patterns are shown for each of the pure CS powder (commercially purchased), the NCS beads, and the Ion-imprinted CS beads (synthesized for this study). There are two observable CS peaks at 2θ = 9° and 2θ = 20°, in some literature these peaks are referred to as common crystals of CS polymeric [83,84]. These characteristic peaks of the CS indicate that the crystalline structure exists in the CS polymer, however, since the CS involves molecules not separate atoms or ions, sharp peaks are not expected to be obtained, and the crystallinity of the CS polymer will depend mostly on the arrangement of the polymer chains rather than the atoms as in most XRD analytics as also reported in Ref. [83]. The presence of the characteristic CS peaks in the XRD patterns of the NCS and the IIC, suggests that the structural arrangement of the CS polymer was intact during the synthesizing process of the beads using the phase inversion method. The broad peaks that appeared in 2θ = 32°- 50° relate to the amorphous region of the chitosan's structure as reported in Ref. [85].

Most polymers show both, crystalline and amorphous regions, as the crystallinity degree of polymers refers to the presence of regions where the polymer chains align with each other, where stereoregularity of the polymer's backbone side groups is required [86]. The effect of the EPI cross-linker on the beads was confirmed by the XRD patterns in Fig. 8B, where the peaks of the crystalline groups were narrower and higher compared to the other modifications. This might be explained by the fact that the EPI binds CS chains with each other through (-OH) groups, leaving behind a more regular structure and more aligned chains leading to a more stereoregularity, which is related to the crystallinity of the samples. The grafting of the CS beads has also affected the X-ray diffraction patterns of the beads, where the intensity of the crystalline beads has decreased as well as their sharpness, meaning that the beads have become more amorphous. The decrease in crystallinity has reported an increase in the adsorption area of the adsorbent [83], which is confirmed by the increase in the IIC-SiO_2_-EPI beads' batch adsorption experiments, where it has achieved higher adsorption capacity compared to the other Ion-imprinted beads.

### 3.2 Polymer molecular characterization

#### 3.2.1 Fourier-transform infrared spectroscopy (FTIR)

**Fig. 9:**
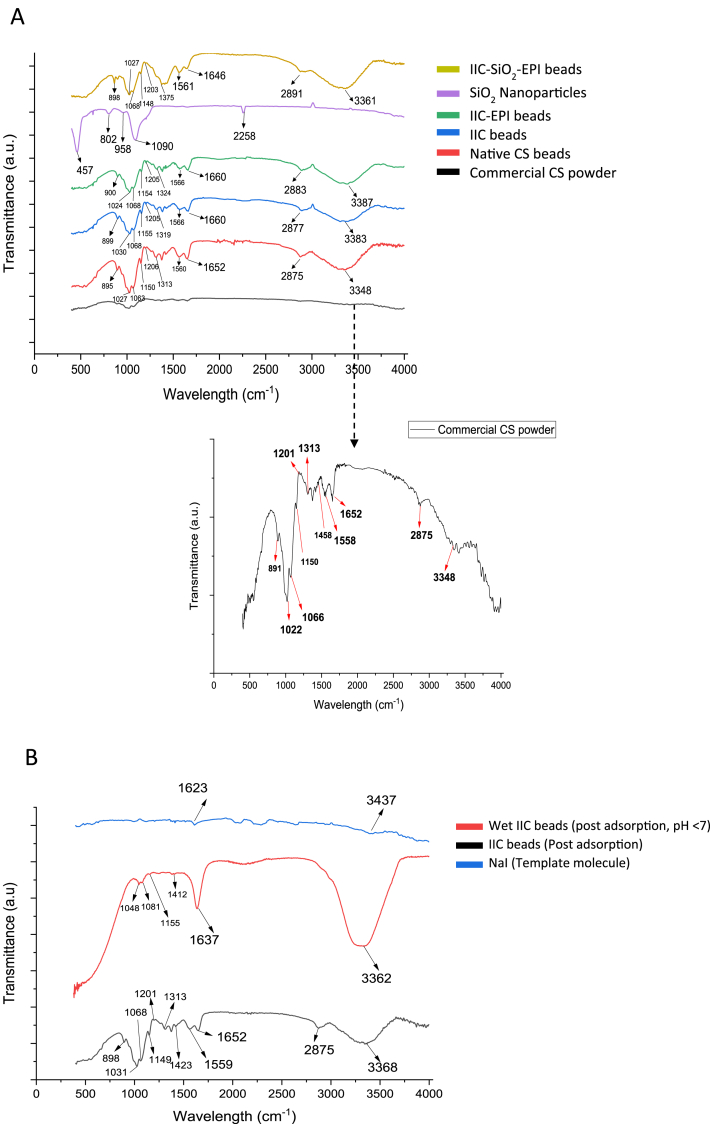
A) FTIR spectra of the commercial CS powder, SiO_2_ nanoparticles, and the dry CS beads at pH 7. B) FTIR spectra of NaI template molecules, dry IIC beads post adsorption, and wet IIC beads post adsorption at pH < 7.

FTIR analysis is one of the most popular techniques used to identify and detect chemical bonds in materials, therefore it was used to understand the adsorption mechanism of the I^−^ ions in the NCS and the IIC beads. FTIR was conducted using Vertex 80v (Bruker). The resulting spectra have shown a broad band of the NCS beads between peaks of 3287 cm^−1^ and 3348 cm^−1^, shown in Fig. 9A, which attributed to the combined effects of (N–H) stretching bond of primary amines and the (O–H) stretching vibrations, respectively. This also indicates the existence of electrostatic exchangeable charges between the alcohol and amine groups as reported in Ref. [87].

The bands 2875 cm^−1^ and 1313 cm^−1^, shown in Fig. 9A, are due to the (C–H) asymmetric stretching vibration in-CH_2_ [88] and the (C–H) bending vibrations in-CH_3_ functional groups, respectively. Other bands were associated with the amide groups in the CS beads, such as the bands in 1652 cm^−1^ and 1558 cm^−1^, and this is where the protonated NH_3_^+^ groups appear if samples were at acidic mediums (pH < 7) [89]. (C–O) stretching vibration peaks also appeared at 1027 cm^−1^ for the NCS and 1030 cm^−1^ for the IIC beads due to the oxygenated groups in the aliphatic ethers of the chitosan. (C–O) stretching vibration peaks also appear in the cross-linked beads, IIC-EPI and IIC-SiO_2_-EPI, at around 1030 cm^−1^, with an increase in the peak transmittance, this increase might be due to the more (C–O) bonds formed during the activation process of the crosslinking with EPI as also reported in Ref. [90]. The peak at 2875 cm^−1^ for the NCS indicates the (C–H) symmetric stretching vibrations of the –CH_2_ groups [88], which also appear IIC, and IIC-EPI beads. The presence of the peaks at the wavelengths 890 cm^−1^ to 947 cm^−1^ in all the samples refers to the aryl group of the CS with 1,4-disubtribution [88], which is also where glycosidic linkage is. There is no detection of iodine-containing bonds, which proves the proper removal of the iodide ion from the CS chains. The appearance of all the characteristic peaks of the chitosan in the prepared beads proves that the ion-imprinting synthesis process does not damage the chitosan organic structure.

The FTIR spectra of the SiO_2_ nanoparticles have shown observable peaks at the region from 457 cm^−1^ to 958 cm^−1^ which indicates the (Si–O) bonds vibrations [91], these peaks were not successfully detected in the modified IIC-SiO_2_-EPI beads due to their overlap with the already existent polymeric peaks. Interacted silanol groups with the polymer's carbonyl groups can form (Si–*O*–C) bonds, which have reported peaks at 1080-1110 cm^−1^ [92], however, the peak was not detected in the formed IIC-SiO_2_-EPI beads, which means that the chitosan gel was physically combined with the SiO_2_ nanoparticle without chemical bonds formation, a similar result was also reported in Ref. [44].

Fig. 9B shows the FTIR spectra of the IIC beads post-adsorption, which didn't reveal any possible chemical bonds between the I^−^ ions and the chitosan's functional groups, and this suggests that chemisorption is not an adsorption mechanism for the ion-imprinted beads. Moreover, no characteristic peaks of the template molecules, NaI, were observed in the prepared ion-imprinted beads. NaI has reported in Ref. [93] a characteristic peak at 1623 cm^−1^ due to the (Na–I) bond and a peak at 3437 cm^−1^ that is associated with the O–H stretching bonds as a result of the water impurities in the salt which also agrees with the NaI FTIR spectra at Fig. 9B. Since the (Na–I) bond was not observed in all the IIC beads' spectra, the NaI particles were removed successfully from the chitosan matrix. In addition, the wet IIC beads have shown higher transmittance values for each of the combined (O–H) and (N–H) peaks, which indicates the higher water content in the pores of the wet CS beads. As mentioned earlier, at pH < 7, the protonated NH_3_^+^ groups start to appear, and this can be noticed in Fig. 9B, where the (N–H) bending bonds' peak at 1637 cm^−1^ transmittance increases. A summary of all the observed peaks, their corresponding intensity, and their interpretation is found in Table 10.

**Table 10:** Bands Obtained in the FTIR data, Their Corresponding Assigned Molecular Bonds, Reported Wavelength, Measured Wavelengths, and their Intensity.

Vibration Type and Assignment a)	Reported Range	Pure Chitosan (commercial)	Native CS Beads	IIC Beads	IIC-EPI Beads	IIC-SiO_2_-EPI beads	IIC beads (post adsorption)	Intensityb)
ν (CH_3_) in R–CH_3_	2885-2860 [92]	2875	2875	2877	2883	2875	2875	s, br
ν (CH_2_)	2936-2915 [92]	2904	2914	2891	2904	2927	2954	vw
ν (OH in water)	3400-3200 [92]	3348	3348	3383	3387	3361	3368	vs
ν (OH) in O=C–OH glucopyranose units	3200-2600 [92]	3110	3114	3126	3311	3292	3294	vw
Secondary amine, τ (N–H)	1695-1630 [92]	1652	1652	1660	1660	1646	1652	s
Primary amine, τ (N–H)	1650-1590 [92]	1558	1560	1566	1566	1561	1559	s
$\delta_{as}$ (CH_3_)	1470-1250 [92]	1313	1313	1319	1324	1375	1313	m
τ (CH_2_)	1475-1445 [92]	1458	1458	1465	1463	1430	1423	vw
δ (–CH_2_–OH)	1300-1050 [92]	1201	1206	1205	1205	1203	1201	w
$\nu_{s}$ (C–*O*–C)	1140-800 [92]	1066	1063	1068	1068	1068	1068	w
ν (C–O)	1020 - 1090 [90]	1022	1027	1030	1024	1027	1031	s
ν (C–N)	1130-1190 [92]	1150	1150	1155	1154	1148	1149	m
δ (C–H) Aromatic ring	804-991 [94] 670-900 [92]	891	895	899	900	898	898	m
δ (C–H) in rings, glycosidic linkage (COC), and δ (CCC)	948-980 [94]	946	945	947	–	–	945	vw
**Possible Interactions**								
Si–*O*–C	1080-1100 [92]	Not detected						
C–I	500-600 [92]	Not detected						
ν (H–I)	2229.6 [95]	Not detected						

a) Vibration assignment: stretching vibrational mode (ν), deformational (δ), bending (τ), and symmetrical (s) and antisymmetric modes (as).

b) Bond intensity: s = strong, m = medium, w = weak, v = very, br = broad.

#### 3.2.2 Raman spectroscopy

**Fig. 10:**
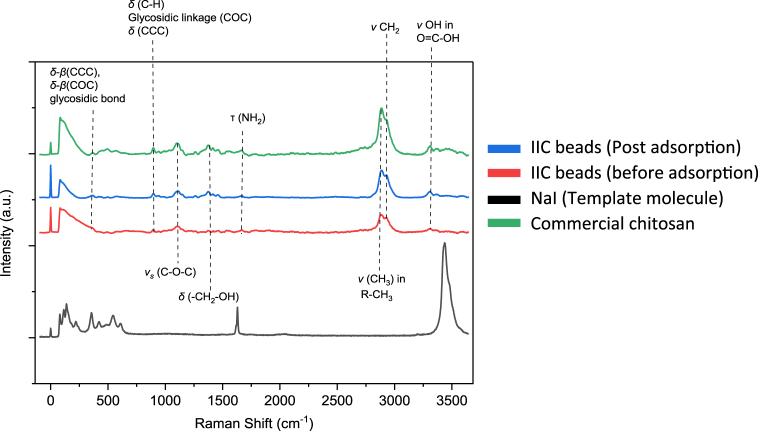
Raman spectra of CS (commercial), pure NaI, and the synthesized CS beads: IIC beads (before-adsorption) and IIC (post-adsorption). Silicon was used for calibration (narrow peak at the ∼0 cm ^−1^ wavelength). (For interpretation of the references to color in this figure legend, the reader is referred to the Web version of this article.)

To obtain Raman spectra of the polymeric samples of this study, alpha300 RA (Witec) was used with a 532 nm excitation wavelength laser through 50 × lenses. The obtained spectra were recorded using 1000 accumulations per sample, with an integration time of 0.5 s. The Raman spectra can help to determine the molecular structure of unknown organic compounds, however, in this study, the commercial chitosan powder's spectrum has been measured at first, as a reference, to compare the prepared beads to and determine the change in the molecular structure of the synthesized samples. Fig. 10 shows the Raman spectra of the commercial pure chitosan powder in comparison with the prepared IIC beads. It is worth mentioning that one of the issues associated with the Raman spectra of the chitosan is the fluorescence associated with the chitosan, which causes a decrease in the intensity of the peaks, therefore, all the peaks were considered, even the very weak ones. Fig. 10 shows baseline-corrected spectra, where the fluorescence effect is neglected.

Comparing the pure CS Raman spectra to all the other adsorbents, it was noted that none of the bonds disappeared from the spectra, meaning that the synthesizing process of the beads didn't cause any damage to the polymer's structure. In Fig. 10, some of the chitosan characteristic bonds were detected better than the FTIR spectra in Table 10, such as the (C–C–C) and the (C–*O*–C) bonds of the chitosan's glycosidic bonds and linkage, which supports the safety of the ion-imprinting procedure to the chitosan chains. Fig. 10 also shows the Raman spectra of the synthesized IIC beads before and post-adsorption, as well as the Raman spectra of the template molecules NaI. The figure shows that the Raman spectra of the IIC beads post adsorption didn't contain any I^−^ related bonds, H–I for instance which has a peak at a wavelength of 2229.6 cm^−1^, or any of the other peaks found in the NaI Raman spectra as shown in Fig. 10 which confirms that the adsorption isn't chemical, as no chemical bonds have been formed due to the batch adsorption of I^−^ from an aqueous solution, this result is also supported by the FTIR of this study.

### 3.3 Thermal characterization

#### 3.3.1 Thermal gravimetric analysis (TGA)

**Fig. 11:**
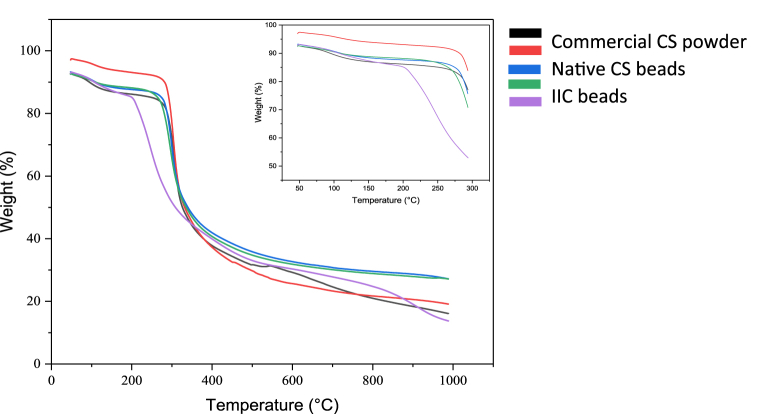
Thermal gravimetric analysis (TGA) of A) pure CS (commercial), B) NCS beads, C) IIC beads, D) IIC-EPI beads, and E) IIC-SiO_2_-EPI beads.

A thermal gravimetric analysis (TGA) was conducted using a thermal analyzer SDT 650 (TA Instruments) to study the changes in the weight of the sample (%) by increasing the temperature from ∼50 °C to 1000 °C. Pure CS powder was tested first, to consider it as a reference to compare the synthesized beads to and identify whether the synthesizing of the beads caused any changes in the thermal stability of the polymer or not. The TGA thermograms in Fig. 11 shows two stages of the weight loss of pure CS. The first stage was in a range of 50 °C–130 °C, with a total waste of weight of around 5 %. Then, the pure CS weight remained almost constant until the second stage, in the second stage a loss of around 62 % occurred where the weight declined as the temperature increased from 272 °C to 774 °C, these results have agreed with other reports [96]. The first stage of weight loss is associated with the loss of water molecules, not the polymer, while the second stage is associated with the primary degradation of the pure polymer at 272 °C, where the samples mostly degraded at a temperature of 774 °C, where only 20 % of the sample's weight is left. The same stages appeared in the synthesized beads samples, NCS, IIC, IIC-EPI, and IIC-SiO_2_-EPI, where all the beads showed higher weight loss in the first stage, mostly the IIC-SiO_2_-EPI samples Fig. 11, which only proofs the higher content of water molecules in the beads. The modification with SiO_2_ has decreased the thermal stability of the samples, where the primary degradation with these samples starts at 196 °C, which is almost 76 °C lower than the pure CS as shown in Fig. 11.

### 3.4 Equilibrium adsorption capacity

Since the adsorbates in this study are aqueous samples with different initial iodide ion concentrations, without the presence of other ions, the homogenous iodide ion samples post-adsorption were quantitatively measured using spectroscopy that follows the Beer-Lambert law [97]. In the Beer-Lambert law, the radiation absorbance of a sample, A, is directly proportional to the product of the molar absorptivity, ε, in (L. mmol^−1^.cm^−1^), path length, b, in (cm), and concentration, C, in (mmol.L^−1^), as shown in equation **(2)**.

(2) A=εbC

**Fig. 12:**
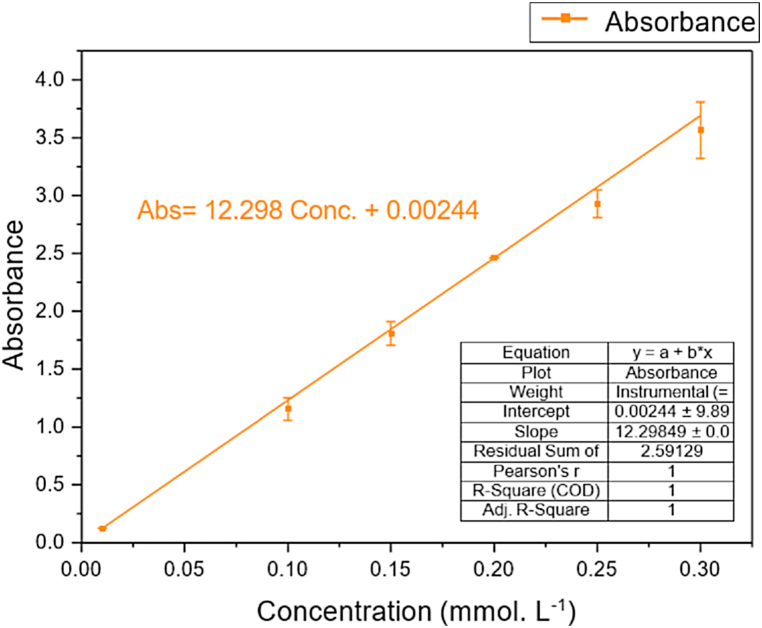
Standard curve of diluted iodide aqueous solutions concentrations measured using UV–vis spectroscopy (UV-1900i Shimadzu).(3)$C_{e}=\frac{Abs-\;0.00116}{12.321}$

The spectroscopy used in measuring the absorbance of the adsorbates after the adsorption was UV–vis spectrometer UV-1900i (Shimadzu). A standard curve that finds the linear relationship between the absorbance and the concentration can help identify the unknown concentration of the samples post-adsorption. In Fig. 12, the standard curve was formed by measuring the adsorption value of different known concentrations of iodide solutions at 226 nm [98], which is the reported iodide ion wavelength. The yielded standard curve gives a linear relationship between concentrations of the iodide ion in the solutions and the absorbance value measured by the UV–vis that can be re-arranged to equation (3) and used to find unknown post-adsorption equilibrium concentration C_e_ in (mmol.L^−1^).

After batch adsorption and knowing the initial concentration of the adsorbate C_0_ in (mmol.L^−1^), then finding the equilibrium concentration using equation **(3)**, the adsorption capacity of the adsorbent which is the quantity of the adsorbate removed by the adsorbent per unit mass or volume of the adsorbent, can be calculated using equation **(4)** [71], where the adsorption capacity Q_e_ in (mmol.g^−1^), V is the volume of the batch adsorption system (L), and m is the adsorbent dosage weight in (g).

(4) $$Q_{e}=\frac{\left(C_{o}-C_{e}\right)V}{m}$$

In addition to adsorption capacity, the adsorption performance can also be expressed in terms of the removal efficiency, where the I^−^ removal efficiency was calculated using equation **(5)**:

(5) $$I^{-}\;\text{Removal}\;\text{Efficiency}\;\left(\%\right)=\frac{C_{0}-C_{e}}{C_{0}}*100$$

### 3.5 Effect of the adsorbents’ modifications

#### 3.5.1 Effect of the cross-linking ratio

**Fig. 13:**
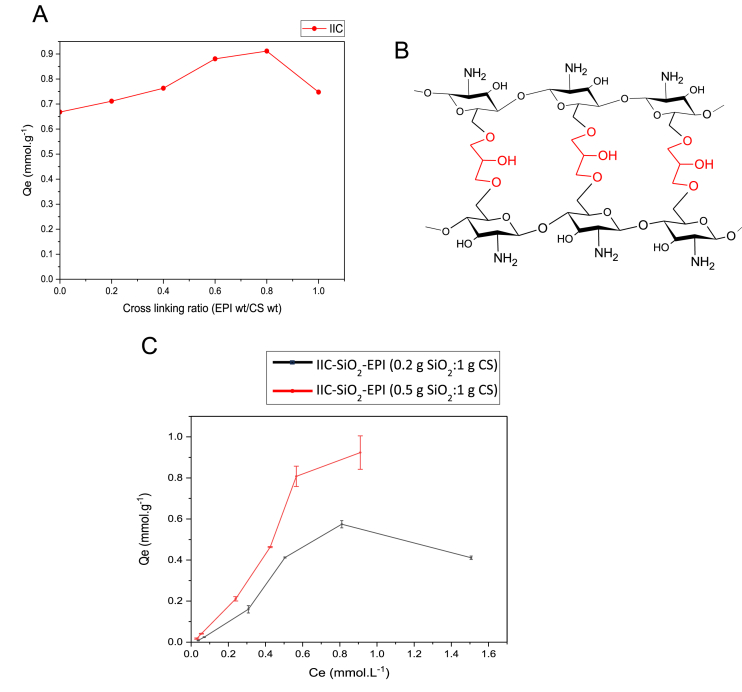
A) Effect of the cross-linking ratio (w/w) of IIC beads on the adsorption capacity. B) The cross-linking mechanism of chitosan with epichlorohydrin. C) Effect of the grafting ratio of the IIC-EPI (g SiO2: g CS) of IIC beads on the adsorption capacity.

The cross-linking ratio is as important as the choice of the cross-linker. Since EPI has been chosen to modify the CS polymeric network, determining the best cross-linking ratio was tested using the experimental conditions in Table 2 to find the best cross-linking ratio for the CS's modification. The adsorption capacities at different cross-linking ratios were measured and plotted in Fig. 13A, epichlorohydrin has shown its ability to increase the absorption capacity until a certain optimum ratio, afterwards, the adsorption capacity starts to decrease. Similar results were reported in Ref. [99]. This behavior might be due to the surface density that the hydrogels exhibit with increasing the cross-linker's ratio. The more the cross-linker is present in the chitosan's network as shown in the illustrative graph in Fig. 13, the more compact the network will be. Denser surfaces are not favorable for porous adsorbents, as they tend to decrease the accessibility of the adsorbates to the pores. Based on this work, an epichlorohydrin weight/chitosan weight (w/w) ratio of 0.8 has shown the best results in terms of the achieved adsorption capacity, therefore, this ratio was used for the preparation of the IIC-EPI beads in the later stages of the study.

#### 3.5.2 Effect of the grafting ratio

Two grafting ratios of 0.5:1 and 0.2:1 gSiO_2_:gCS were tested, through batch adsorption experiments. Fig. 13C shows the difference in the performance of the two grafting ratios, where the lower grafting ratio of (0.2:1) g SiO_2_:g CS has shown a better performance than the (0.5:1) g SiO_2_:g CS grafting ratio. Since the FTIR spectra of the cross-linked IIC-SiO_2_-EPI beads in Fig. 9A confirm that the chitosan gel is only mixed with the SiO_2_ nanoparticle without a chemical bond, the decrease in the SiO_2_ content in the CS gel makes the gel less dense, which is a desirable texture for making hydrogel beads. In the studies [100,101], porosity increase was reported with increasing the solvent of acetic acid concentration and decreasing the polymer solute concentration in the polymer's gel. This supports explaining this study's increase of adsorption performance with the decrease in the SiO_2_ nanoparticles which is also a solute in this polymeric gel. Based on these results, the grafting ratio (0.2:1) g SiO_2_: g CS was used in the preparation of the IIC-SiO_2_-EPI beads used later in this study.

### 3.6 Effect of adsorbent dosage

**Fig. 14:**
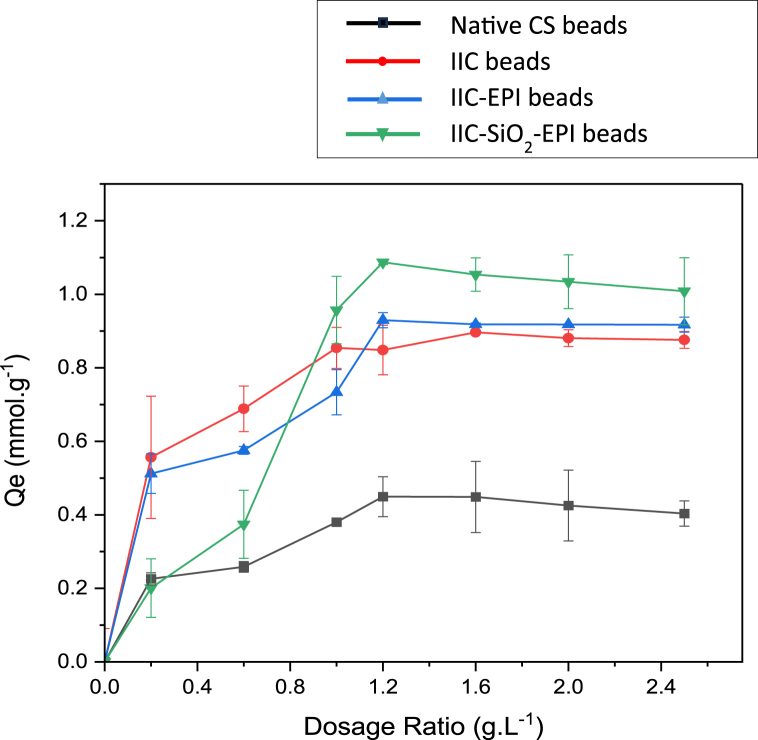
Effect of the adsorbent dosage (g/L) of the NCS beads, IIC beads, IIC-EPI beads, and IIC-SiO_2_-EPI beads on adsorption capacity.

To investigate the effect of the adsorbent dosage on the I^−^ removal efficiency, adsorbents at different dosages (10–125 mg) were dispersed to a controlled aqueous solution volume of 50 mL, achieving dosage (mass of adsorbent/volume adsorbate) ratios of (0.2–2.5 g/L). The adsorbent dosage ratio versus the adsorption capacity is shown in Fig. 14. In Fig. 14 the increase of the adsorbent dosage has increased the I^−^ removal efficiency due to the increase of the active sites of the pores and the adsorption contact surfaces until a dosage ratio of (1–1.2 g/L) for each of the iodide imprinted beads with/without the modifications, where the adsorption capacity reaches a plateau with all of the samples, and this is common in microporous adsorbents, where the micropores tend to be clogged with the iodide ions, so the ions won't be able to diffuse to inner pores, regardless of the number of the beads used, especially if the pores have heterogeneous surfaces that do not all show the same number of pores. Thus, 50–60 mg of the beads was the proper dosage to attain the highest adsorption capacity for all of the samples.

### 3.7 Effect of adsorption speed

**Fig. 15:**
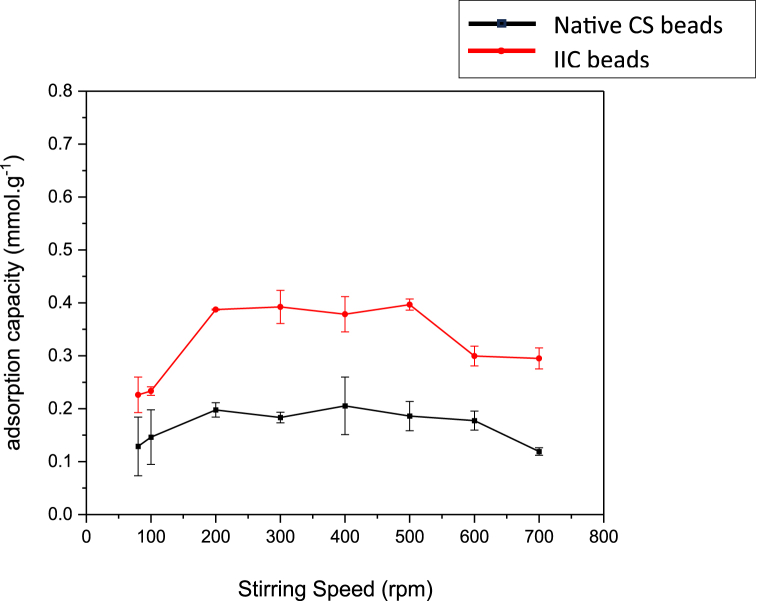
Effect of stirring speed on NCS and IIC beads on adsorption capacity.

The effect of the applied stirring speed is shown in Fig. 15A and B. The adsorption capacity has increased rapidly with increasing the speed of stirring from 80 rpm to 200 rpm, and reached an average maximum adsorption capacity with slight decreases due to possible experimental error at the stirring conditions (200–500) rpm, which at low stirring speed, the beads tend to stay at the surface of the solution, achieving a lower contact area of the beads and the solution compared to higher speeds. High stirring speeds don't allow for the ions to diffuse through the inner pores of the beads, where ions enter the surface pores very quickly causing them to block the micro pores faster achieving a decrease in adsorption capacity (600–700) rpm. The results have shown a similar pattern for each of the NCS and the IIC beads meaning that the imprinting doesn't influence the behavior of the beads at different stirring conditions.

### 3.8 Effect of pH

Reportedly, chitosan has a pK_a_ value of 6.3–7.0 [37], and hence in lower pH medium or acidic solutions, the hydrogen atoms (H^+^) content can protonate the-NH_2_ groups in the chitosan chains to-NH_3_^+^, as the reaction expressed in equation **(6)** [102], R′ is the chitosan's chain where the NH_2_ group binds.

(6) $$R^{\prime }-NH_{2}+H^{+}⇌R^{\prime }-NH_{3}^{+}$$

**Fig. 16:**
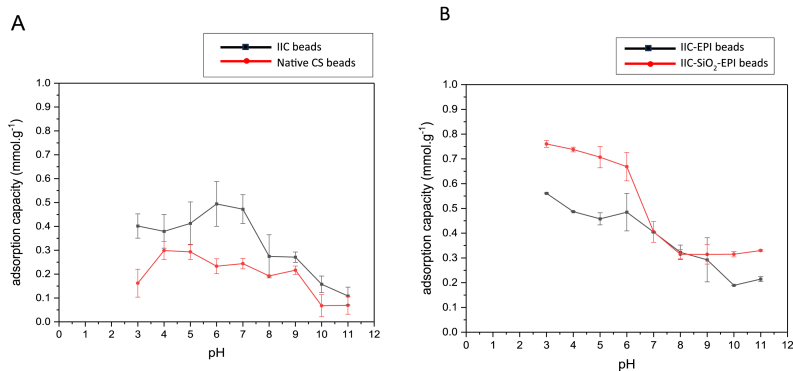
Effect of the solution pH of the A) NCS beads and IIC beads, B) IIC-EPI beads, and IIC-SiO_2_-EPI beads on adsorption capacity.

Therefore, at lower pH mediums, electrostatic interactions between the adsorbate anions and the positively charged chitosan surface increase, which also leads to the increase of the chitosan's adsorption capacity. At higher pH values and alkali solutions, the hydroxyl ions (OH^−^) will increase and compete with anionic adsorbates on the chitosan's adsorption sites, leading to a decrease in the adsorption capacity [103]. In Fig. 16A and B, all the samples have shown an increase in the adsorption capacity at lower pH and an overall decreasing pattern in the adsorption capacity at higher pH values. Visually, the iodide-imprinted chitosan beads, at acidic mediums, molecularly imprinted IIC, IIC-EPI, and IIC- SiO_2_-EPI, have shown more swelling than the NCS beads, where the NCS beads have shrunk at acidic pH values and dissolved even completely at pH of 3. This happens due to the catanionic nature of the chitosan at lower pH values, where the-NH_2_ groups protonate to-NH_3_^+^, the ionization of these functional groups along the chitosan's backbones causes an electrostatic repulsion inside the chitosan's network [104,105]. Since this study deals with hydrogel beads, it's very common to notice a swelling due to the electrostatic repulsion between the protonated amino groups in the chitosan, and since the imprinted beads have more pores and cavities that are exposed to the acidic medium compared to the non-imprinted hydrogel beads, the imprinted beads are more likely to swell. There was no evidence of the polymer's distortion, as returning the beads to neutral mediums caused the return of the beads to their original state.

The cross-linked beads in Fig. 16B have shown more mechanical stability at lower pH values compared to the non-cross-linked beads in Fig. 16A. The highest adsorption capacity for cross-linked samples has been achieved at a pH of 3, where chitosan tends to be completely protonated at pH < 3 [106], however, due to the deforming in the non-cross-linked beads at an extremely low pH value, the beads were tested until pH 3, as the purpose of the study is to find the operating conditions where chitosan performance is enhanced not deformed. It is noticed in Fig. 16A, that the absorption capacities of the non-cross-linked beads decrease at pH < 5, and this is due to the dissolving of the wet beads where the adsorbed iodide ions are released back from the pores to the adsorbate solution. Therefore, cross-linking with epichlorohydrin is required in the treatment of acidic nuclear wastewater.

### 3.9 Effect of Co-existing ions

**Fig. 17:**
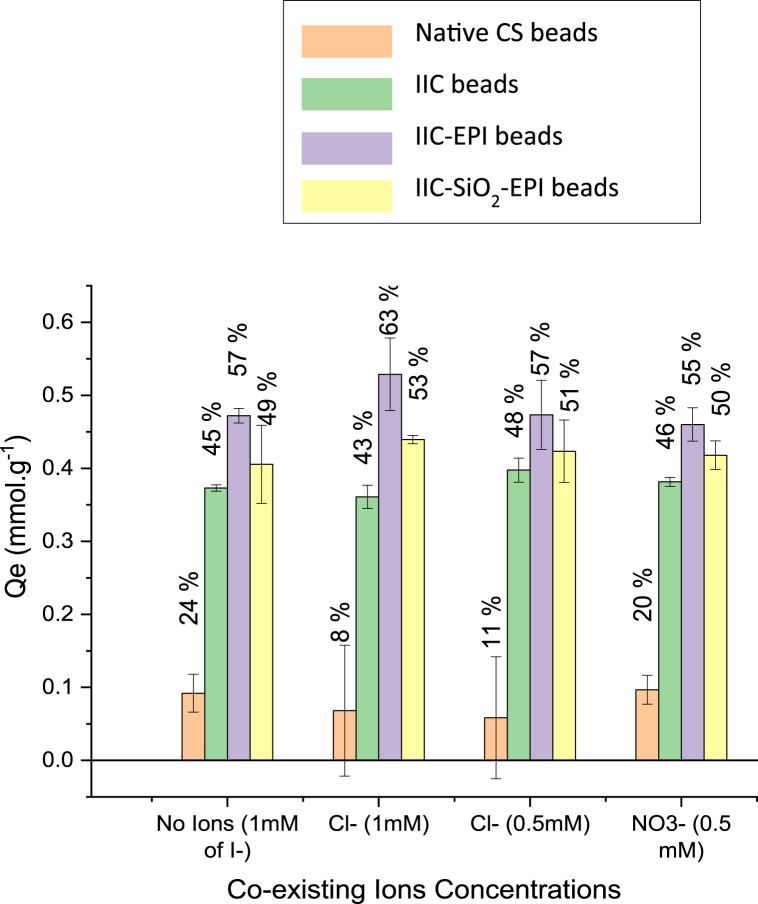
Effect of the co-existing ions of the NCS beads, IIC beads, IIC-EPI beads, and IIC-SiO_2_-EPI beads on adsorption capacity and the corresponding adsorption efficiency.

High salt concentrations have been associated with high ionic strengths that compete with the targeted adsorbate ions and lower the adsorbent's capacity [[107], [108], [109]], at which the salt counter ions surround the adsorption sites and lead to reducing their affinity to the targeted ions. For the iodide imprinted beads; IIC, IIC-EPI, and IIC-SiO_2_-EPI, there was no notable reduction in the I^−^ adsorption capacities with the presence of these ions as shown in Fig. 17, while the non-imprinted NCS beads have shown a major decrease of 67 % and 57 % in the adsorption capacity with the co-existence of 1 mΜ of KCl and 0.5 mΜ of KCl, and a slight decrease of 18 % in the adsorption capacity due to the existence of AgNO_3_ in a concentration of 0.5 mΜ, while the co-existence of 1 mΜ AgNO_3_ in the adsorbate caused the I^−^ peak to not be detected by the UV–vis spectrum, and this is due to the equal concentrations of the Ag^+^ and I^−^ ions that cause the formation of AgI covalent bonds which precipitates as yellow precipitates and leaves an undetectable amount of the I^−^ ion in the solution.

### 3.10 Adsorption kinetics and isotherms

#### 3.10.1 Adsorption kinetics

Adsorption kinetics are investigated for the sake of determining the relationship between the adsorption capacity at a specific time (Q_t_) and the time (t), however, it can also deliver insight into the sorption mechanism [110]. The most common adsorption kinetic models used are the Lagergren pseudo-first-order (PFO) and pseudo-second-order models (PSO) of adsorption. PFO assumes that the adsorbate rate of uptake is directly proportional to the difference between the saturation or equilibrium adsorption capacity and time (t), which is also proportional to the amount of effective adsorption sites. This model is usually applicable over the initial stages of the adsorption processes, the first 20–30 min [111]. Kinetics of an adsorption process have been commonly fitted pseudo-first-order models when the diffusion is controlled by physisorption [112]. The PSO assumes that the overall adsorption is controlled by the rate of diffusion of the adsorbate into the adsorbent's pores [113,114], where in most cases the rate-limiting step is chemical sorption. The PSO models have shown their ability to predict the behavior of the adsorption process over the whole range of the adsorption [111], therefore, the adsorption rate is dependent on the adsorption capacity not the concentration of the adsorbate such as in processes where chemisorption occurs alone [115].

The relationships have been explored using the PFO and PSO kinetic equations, expressed in equations **(7)** and **(8)** [71]:

(7) $$Q_{t}=Q_{e}-Q_{e}*e^{-k_{1}t}$$

(8) $$Q_{t}=\frac{Q_{e}^{2}\;k_{2}t}{1+Q_{e}k_{2}t}$$

In equations **(7)** and **(8)**, Q_t_ and Q_e_ are in (mmol.g^−1^), t (min), k_1_ is a first-order constant (min^−1^), and k_2_ is a second-order constant (g.mmol^−1^.min^−1^). The correlation coefficient (R^2^) and the calculated equation parameters are tabulated in Table 11. The linear forms of the PFO and PSO kinetic equations are mentioned in equations **(9)** and **(10)**:

(9) $$\text{In}\left(Q_{e}-Q_{t}\right)=\ln\left(Q_{e}\right)-\frac{k_{1}\;t}{2.303}$$

(10) $$\frac{t}{Q_{t}}=\frac{1}{Q_{e}^{2}k_{2}}+\frac{t}{Q_{e}}$$

**Table 11:** Constants of non-linear pseudo first and second order models of I^−^ ions adsorption.

Adsorbent	pH	Q_e_a) [mmol.g^−1^]	Pseudo-First-Order			Pseudo-Second-Order		
			Q_e_b) [mmol.g^−1^]	k_1_ [min^−1^]	R^2^	Q_e_ [mmol.g^−1^]	k_2_ [g mmol^−1^. min^−1^]	R^2^
Native CS	7	0.1512	0.1472	1.0144 × 10^13^	0.8950	0.1533	4.9615	0.9056
IIC	7	0.2416	0.2492	0.1792	0.9426	0.2641	1.2334	0.9661
IIC-EPI	7	0.4196	0.4323	0.0758	0.945	0.4745	0.2438	0.9722
IIC-SiO_2_-EPI	7	0.5130	0.5146	0.2632	0.9883	0.5185	3.5999	0.9861
IIC-SiO_2_-EPI	5	0.6944	0.6702	0.1570	0.9835	0.6997	0.5397	0.9945

a) Experimental Data.

b) Calculated data by the model.

**Fig. 18:**
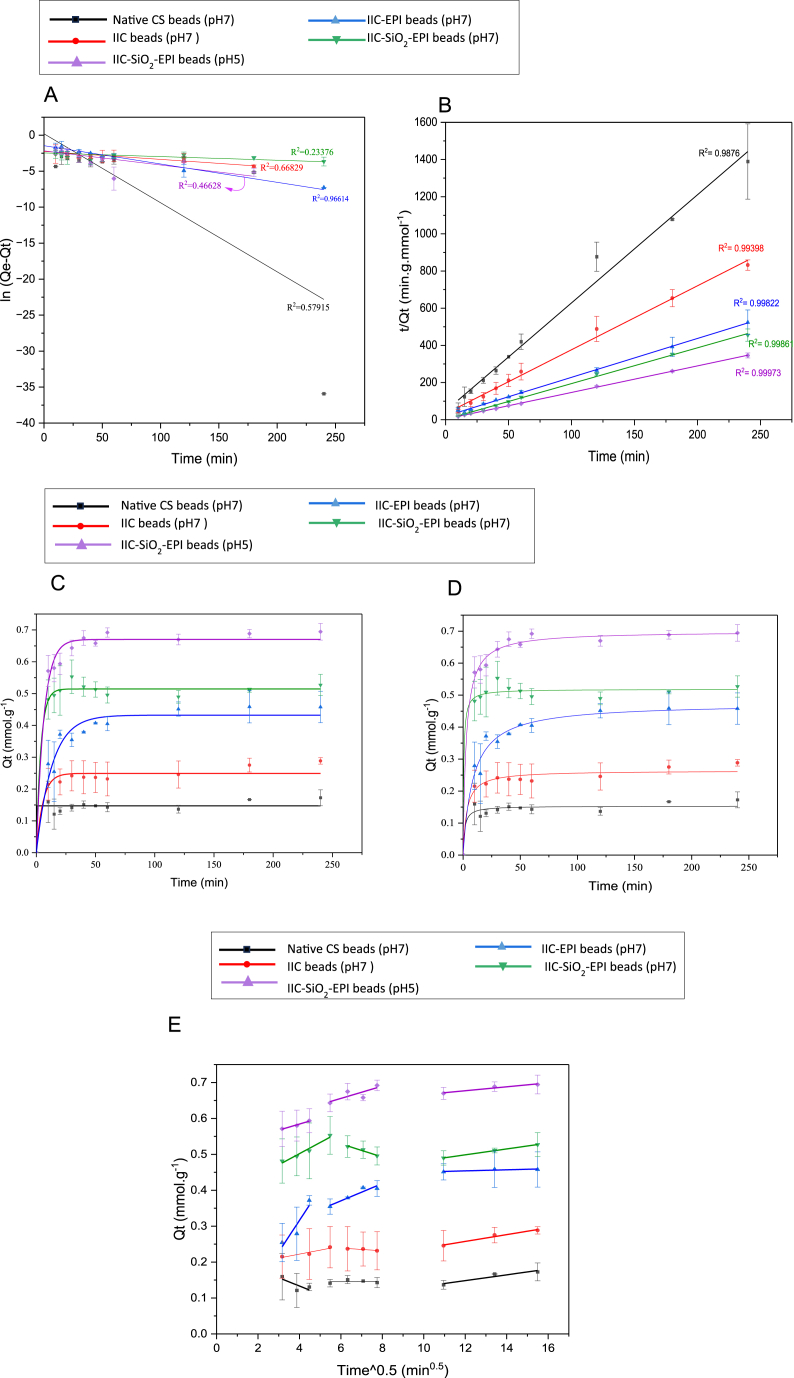
Native CS (pH 7), IIC (pH7), IIC-EPI (pH 7), IIC-SiO2-EPI (pH7), IIC-SiO2-EPI (pH 5) fitted with A) Linear Fitting of PFO model B) Linear Fitting of PSO model C) Non-linear Fitting of PFO model D) Non-linear Fitting of PSO model and E) Weber and Morris intra-diffusion model.

Fig. 18A and B show the linear fittings of the experimental data of the PFO and PSO relationships, respectively. It is seen that the linear PSO kinetic model gives better fitting compared to the PFO kinetic model with a goodness of fit value of (R^2^ = 0.988 for the NCS beads and >0.990 for the rest of the samples). Besides the interpretation of PSOs to chemisorption, PSO can also be interpreted that the adsorption rate is proportional to the available adsorption sites that are largely controlled by electrostatic attraction between the negative I^−^ ions and the positive chitosan surface's pores, which agrees with [116]. According to the non-linear PFO models in Fig. 18C and PSOs models in Fig. 18D, notably, most of the adsorption occurred in the first 30 min for both bead types, however, the IIC has shown higher adsorption capacity compared to the NCS beads. Table 11 summarizes the constants of non-linear PFO and PSO models of I^−^ ions adsorption.

In some cases, MIP adsorbent adsorption can have multiple stages; instantaneous adsorption, intra-particle adsorption, and pore diffusion. To have a deeper insight into the adsorption mechanism occurring, Weber and Morris adsorption kinetics were used to fit the experimental data [115]. Weber and Morris are investigated by plotting the Q_t_ against the square root of the time (t^0.5^) in (min^0.5^) in equation **(11)**:

(11) $$Q_{t}=k_{\mathrm{int}}\;t^{\frac{1}{2}}+C$$

Where the rate constants k_int_ (mmol.g^−1^.min^−0.5^) and C (mmol.g^−1^) are evaluated from the slope and intercept of the regression line [117]. A straight line of the Weber and Morris model suggests that intraparticle diffusion is the sole rate-controlling mechanism, however, a non-zero C value usually indicates the involvement of other mechanisms alongside the intraparticle diffusion (external mass transfer) [118]. Commonly, the Weber and Morris model is not one linear curve, as it usually has two or three linear regions following each other, indicating the different stages of the adsorption mechanisms [119].

**Fig. 19:**
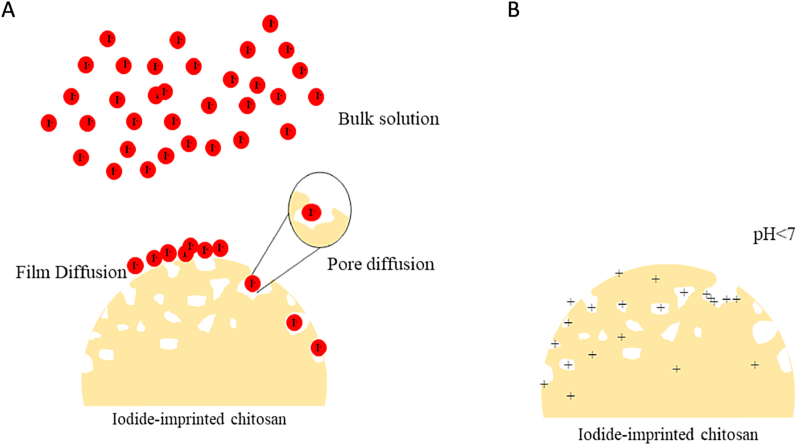
Illustrative figure of A) pore intra-particle diffusion for this study and B) The protonation of the functional groups in chitosan surface/pores.

According to Fig. 18E, the Weber and Morris models were fitted to the experimental data of all the synthesized beads of this study. It is noticed that at the first stage (10–20 min), the adsorption is incredibly fast with a higher positive gradient for all the samples, this indicates the instantaneous adsorption due to the transport of the iodide ions from the bulk solution to the adsorbent's surface. The second stage (30–60 min) shows gradual adsorption, which is called the intra-particle diffusion stage or also called the film diffusion stage as a film layer of adsorbate is formed on the adsorbent's surface [115]. During this stage, the ions tend to enter the pores of the beads, where the pore diffusion will take place in the upcoming stage, it is noticed that at pH 7, all of the beads have shown an almost zero slope at this stage, where the Q_t_ remains constant or slightly decreases with the time, this indicates that most of the ions adsorbed in the first stage were not successfully able to enter the boundary layer, or find the appropriate cavities on the surface to enter the pores. In the last stage, pore diffusion occurs, where the Q_t_ increases slightly and slowly over a longer period of (60–240 min). The lowest of the slope values ($k_{\mathrm{int}}$), usually indicates the slowest step among the adsorption stages, which also indicate the rate-limiting step [117]. The $k_{\mathrm{int}}$ values of the three stages have shown that the $k_{\mathrm{int}}$ is the smallest for each of the second and third stages which all indicate the intraparticle pore diffusion. All the stages of adsorption discussed are shown in the illustrative figure in Fig. 19A.

Table 12 shows the constant values for each of the adsorption stages of all the beads. Lowering the pH, such as pH 5, affects the second phase adsorption capacity, where the adsorption tends to increase unlike pH 7 with the rest of the other beads, this verifies that the protonation of the NH_2_ groups of the chitosan's surface increases the attraction of the negative I^−^ ions to the pores and enhances the pore diffusion Fig. 19B, where the maximum adsorption capacity in the first stage does not decrease in this case. These results come into agreement with [119,120], which all involve adsorption mechanisms related to MIP adsorbents.

**Table 12:** Rate constants for the intraparticle diffusion kinetic model of the I^−^ ions adsorption.

Adsorbent	pH	K _int_ [mmol.g^−1^.min^−0.5^]			C [mmol.g^−1^]			R^2^		
		Stage I	Stage II	Stage III	Stage I	Stage II	Stage III	Stage I	Stage II	Stage III
**Native CS**	7	−0.0230	0.0003	0.0080	0.2254	0.1436	0.0519	0.5694	0.0057	0.8983
**IIC**	7	0.0111	−0.0037	0.0095	0.1778	0.2615	0.1439	0.8935	0.8099	0.9724
**IIC-EPI**	7	0.0879	0.0241	0.0015	−0.0354	0.2262	0.4361	0.8669	0.9000	0.7171
**IIC-SiO2-EPI**	7	0.0311	−0.0183	0.0082	0.3777	0.6388	0.4001	0.9594	0.9527	0.9996
**IIC-SiO2-EPI**	5	0.0170	0.0171	0.0055	0.5164	0.5532	0.6116	0.9733	0.6325	0.9420

#### 3.10.2 Adsorption isotherms

**Fig. 20:**
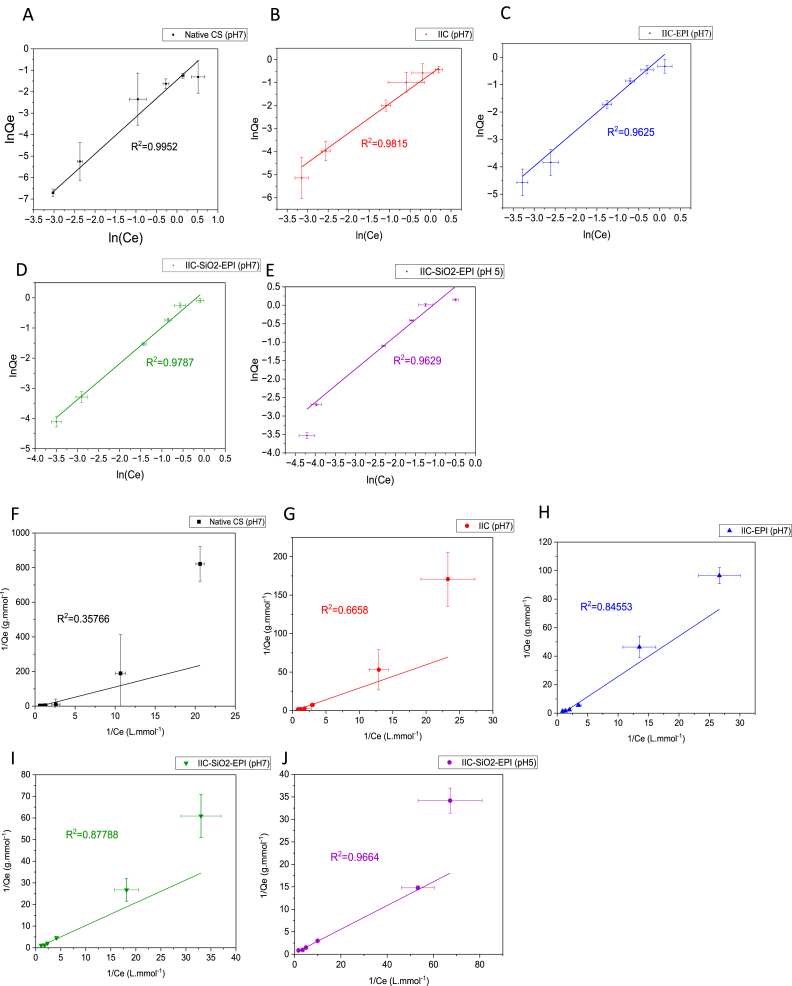
Fitting of Linear Freundlich model to the experimental data for each of the A) NCS (pH 7), B) IIC (pH7), C) IIC-EPI (pH 7), D) IIC-SiO_2_-EPI (pH7), E) IIC-SiO_2_-EPI (pH 5). Fitting of Linear Langmuir model to the experimental data for each of the F) NCS (pH 7), G) IIC (pH7), H) IIC-EPI (pH 7), I) IIC-SiO_2_-EPI (pH7), J) IIC-SiO_2_-EPI (pH 5).

To obtain the data for the adsorption isotherms, batch adsorption experiments considering the parameters in Table 8. Experiments were done in triplicates, where the average values of the adsorption capacities were calculated alongside the standard deviations and the error bars. The obtained experimental data of several adsorption processes were fitted with the linearized Langmuir model (Lineweaver–Burk) [121] and the linearized Freundlich model in equations **(12)** and **(13)**, respectively. Each of the linear Freundlich and the linear Langmuir are shown in Fig. 20A–E and Fig. 20F–J, respectively.

(12) $$\frac{1}{Q_{e}}=\frac{1}{Q_{m}}+\frac{1}{Q_{m}K_{L}}\frac{1}{C_{e}}$$

(13) $$\ln\left(Q_{e}\right)=\ln\left(K_{F}\right)+\left(\frac{1}{n}\right)\ln\left(C_{e}\right)$$

**Fig. 21:**
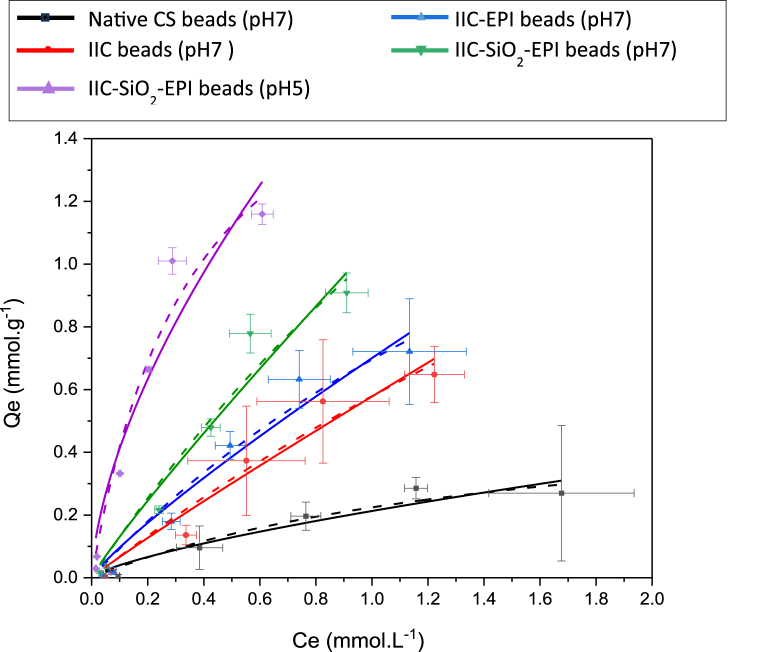
Fitting of Non-linear Freundlich (─) and Langmuir (----) models to the experimental for each of the NCS (pH 7), IIC (pH7), IIC-EPI (pH 7), IIC-SiO_2_-EPI (pH7), and IIC-SiO_2_-EPI (pH 5).

Where K_L_ is in (L.mmol^−1^), and K_F_ is in (L^1/n^·mmol^1−1/n^·mmol^−1^), as n is the Freundlich model empirical coefficient, other calculated parameters, and data are tabulated for the non-linear isotherm models in Table 13. The non-linear Langmuir and Freundlich models are fitted using equation **(14)** and equation **(15)**, respectively, and shown in Fig. 21:

(14) $$Q_{e}=\frac{K_{L}Q_{m}C_{e}}{1+K_{L}C_{e}}$$

(15) $$Q_{e}=K_{F}C_{e}^{\frac{1}{n}}$$

**Table 13:** Constants of non-linear Langmuir and freundlich isotherms of I^−^ ions adsorption.

Adsorbent	pH		Langmuir isotherm				Freundlich isotherm		
			Q_m_ (mmol/g)	K_L_ (L/mmol)	R^2^		K_F_ (L^1/n^·mmol^1−1/n^·mmol^−1^)	n	R^2^
Native CS	7		0.5750	0.6395	0.9526		0.2128	1.3781	0.9239
IIC	7		3.5581	0.1942	0.9600		0.5778	1.0625	0.9548
IIC-EPI	7		2.4593	0.3945	0.9674		0.7003	1.1611	0.9564
IIC-SiO_2_-EPI	7		4.2247	0.3198	0.9600		1.0599	1.1005	0.9537
IIC-SiO_2_-EPI	5		1.9443	2.7366	0.9668		1.7172	1.6135	0.9300

Freundlich isotherm models are generally used to describe physical adsorption that occurs in multi-layers where the amount of the adsorbed molecules increases gradually with the layers. On the other hand, Langmuir isotherm describes monolayer adsorption on a homogenous surface with senergetically equivalent adsorption sites [108,122,123]. The goodness-of-fit ranges of R^2^ were found to be ranging (0.3577–0.9664) and (0.9665–0.9952), for the linear Langmuir and the Freundlich model, respectively, shown in Fig. 20. Which confirms that the Freundlich linear model fits the experimental data better.

The adsorption mechanism of the hydrogel beads studied in this work, combining the kinetic and isotherm models, is a physical intra-particle pore diffusion combined with film diffusion on a heterogenous surface with the possibility of adsorption with few layers inside the beads as it fits Weber and Morris model as well as the Freundlich model.

The heterogeneity of the surface of the beads could explain the higher difference in the adsorption performance of the beads with the three different trials of the experiment, and this is considered a drawback of the phase-inversion synthesis method of the beads. Based on Fig. 21, the adsorption of the IIC-SiO_2_-EPI has given the best performance compared to the other synthesized beads and even better with a pH of 5. This can be explained by the protonation of the NH_2_ groups in the chitosan's matrix which increases the positivity of the adsorbent's pores and the attraction of the I^−^ ions as well. The averaged maximum adsorption capacities recorded at pH 7 for each of the NCS, IIC, IIC-EPI, and IIC-SiO_2_-EPI beads were 0.2854, 0.6477, 0.8541, and 0.9082 mmol g^−1^, respectively, which indicates that the iodide imprinted chitosan beads can achieve almost double the adsorption capacity of the non-imprinted chitosan beads and the modification with epichlorohydrin and grafting with SiO_2_ nanoparticles have an increased effect on the porosity and adsorption of the beads. The IIC-SiO_2_-EPI beads have achieved a maximum adsorption capacity of 1.1594 mmol g^−1^ at pH 5 which is much higher than what is achieved at pH 7 of the IIC-SiO_2_-EPI, which also supports the previously founded results related to the protonation of the chitosan beads‘ surface, so more I^−^ ions are attracted to the pores and adsorbed.

### 3.11 Recyclability and desorption studies

**Fig. 22:**
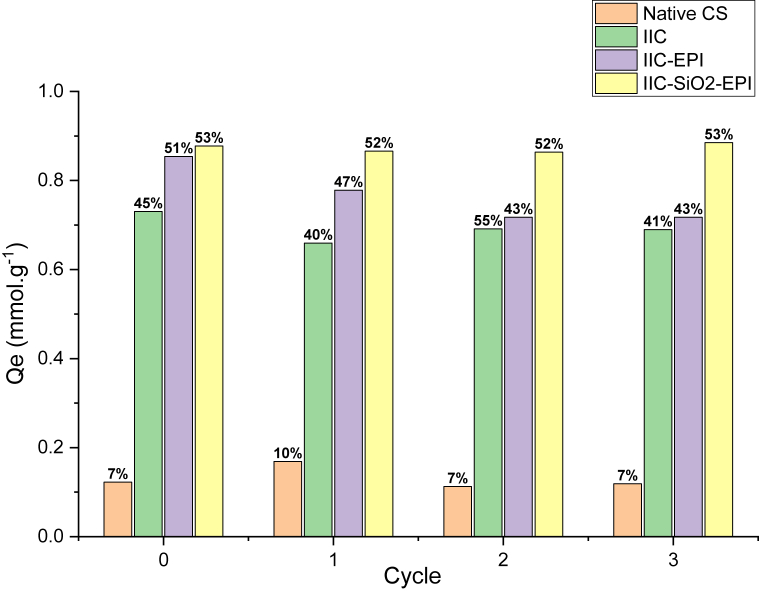
Adsorption capacity and adsorption efficiency of NCS beads, IIC beads, IIC-EPI beads, and IIC-SiO_2_-EPI beads for three cycles of adsorption-desorption.

The recyclability of the synthesized beads was tested at batch adsorption experiments of C_0_ of 2 mmol L^−1^, a pH of 7, an optimum dosage of each sample, and at room temperature. The desorption of the used beads was conducted in a similar manner to the removal of I^−^ ions in the first place via solvent desorption with NaCl eluent solution. Fig. 22 shows three cycles of adsorption-desorption. Ion-imprinted beads have shown better recyclability results, where the adsorption efficiency has an average decrease of 6 % which is satisfactory due to the long process of desorption. Non-imprinted NCS beads have shown similar recyclability to the imprinted beads, indicating that recyclability is also a property of the chitosan polymer.

### 3.12 Comparison with recent studies

The performance of the adsorbents prepared in this study compared with other recent applications of adsorbents for iodide ion (I^−^) removal are summarized in Table 14. Bio-based adsorbents have shown their superiority in availability, natural origin, selectivity, and shorter duration of reaching maximum adsorption, which gives them a natural advantage compared to the other adsorbents. The best-performing beads in this study (IIC–SiO_2_-EPI) have achieved a maximum adsorption capacity of 0.91 mmol g^−1^ at pH 7, this can be considered a moderate adsorbing capacity compared to the published data in Refs. [9,124], which achieved adsorption capacities of 1.10 and 2.30 mmol g^−1^, respectively, however, they required adsorption durations of 600 and 110 min that are much higher than the imprinted chitosan adsorption duration which is only 30 min.

**Table 14:** Adsorbents used for Iodide Ion Removal from Aqueous Solutions.

Adsorbent	Dose (g.L^−1^)	pH	Timea) (min)	Adsorption capacity (mmol.g^−1^)	Refs.
Cross-linked chitosan microspheres	0.5	7	40	0.88	[71]
Calcium alginate–silver chloride composite	5	7	600	1.10	[9]
Ag_2_O grafted sodium niobate nanofibers	0.1	9	110	2.30	[124]
Ag@Cu_2_O nanoparticles	1	7	720	0.20	[125]
Chitosan/hydrophobic-modified montmorillonite	1	7	40	0.30	[126]
Black carbon	5	3	10,080	0.08	[127]
Native CS beads	1.6	7	30	0.29	This work
Iodide-imprinted CS	1.2	7	30	0.65	This work
Iodide-Imprinted CS-EPI	1.2	7	30	0.85	This work
Iodide-Imprinted CS-EPI-SiO_2_	1.2	7	30	0.91	This work
Iodide-Imprinted CS-EPI-SiO_2_	1.2	5	30	1.16	This work

a) Duration of achieving the maximum adsorption.

## 4 Conclusion

The current work represents the synthesis and characterization of IIC beads for removing iodide ions from aqueous solutions, as a proposed solution for the selective adsorption of radioiodine from nuclear wastewater. Morphological characterization such as SEM has revealed the appearance of a more porous surface in the imprinted chitosan beads compared to the smoother surface of the non-imprinted chitosan beads. Molecular characterization, FTIR, and Raman spectroscopy have revealed that no chitosan chains or functional groups were damaged during the imprinting process. At pH 7, each of the NCS, IIC, IIC-EPI, and IIC-SiO_2_-EPI beads have achieved maximum adsorption capacities of 0.2854, 0.6477, 0.8541, and 0.9082 mmol g^−1^, respectively. The IIC-SiO_2_-EPI beads have achieved an adsorption capacity of 1.1594 mmol g^−1^ at pH 5, which indicates the electrostatic attraction of the protonated chitosan beads’ surface. Only cross-linked samples with EPI have withstood lower pH conditions which proves the modification EPI has on chitosan chains. Iodide imprinted beads have shown high selectivity for iodide ions, while the NCS beads have shown a major decrease of >57 % in the adsorption capacity with the co-existence of Cl^−^ ions. Kinetic data fitted the PSO model and Weber and Morris model promoting an intraparticle pore diffusion adsorption mechanism influenced by the electrostatic physical attractions. Desorption experiments have demonstrated excellent reusability of all the chitosan beads.

## Author contributions

Yassmin Handulle Ismail: Writing – original draft, Methodology, Formal analysis, Data curation, Conceptualization. Maryam Al Shehhi: Writing – review & editing, Validation, Supervision, Project administration, Funding acquisition, Formal analysis, Conceptualization. Kean Wang: Visualization, Supervision, Funding acquisition, Formal analysis, Conceptualization. Ali AlHammadi: Writing – review & editing, Visualization, Validation, Supervision, Resources, Project administration, Funding acquisition, Formal analysis

## Data availability statement

Data included in article/supp. material/referenced in article.

## Declaration of competing interest

The authors declare that they have no known competing financial interests or personal relationships that could have appeared to influence the work reported in this paper.

## Figures and Tables

**Table A1:** Traditional Separation procedures used for Nuclear Wastewater Treatment.

Treatment Method	Applications	Procedure	Advantages	Drawbacks	Reference
Evaporation	Low level radioactive effluents with high salt content and heterogenous chemical composition.	Evaporating water leaving behind non-volatile components such as salt and radionuclides.	Very simple process with the volume reduction results.	Possibility of corrosion, scaling, or foaming.	(Efremenkov, 1989)
Chemical Precipitation	Low activity effluents with high salt and mud contents.	Separation of radionuclides by either precipitation, coagulation, or adsorption by insoluble compounds.	Relatively advanced suspended particles removal.	Requires combination with other methods due to its low decontamination results.	(Efremenkov, 1989; Osmanlioglu, 2018)
Solid-Phase Separation	Remove settled or suspended solid contents.	Removal of solid matters via separation equipment (e.g., filters, centrifuges, and hydrocyclones).	Typically, it removes particles down to submicron sizes.	Separation equipment might get exhausted and need replacement.	(Efremenkov, 1989)
Ion Exchange	Efficient for waste solutions with low solids and salts concentrations, with radionuclides that are available in their ionic form.	An ion-exchange material filled fixed bed where contaminated effluent passes through from bottom to top or vice versa.	Ion exchange materials use its active sites to adsorb ions, which can be regenerated after saturation.	A semicontinuous process that requires maintenance efforts (e.g., regeneration, flushing, and refilling operations).	(Efremenkov, 1989; Lehto and Harjula, 1999)

**Table A2:** Summary of the Biopolymeric Adsorbents used for Wastewater Treatment.

Biopolymer	Advantages	Drawbacks	References
Chitosan	1. Second most abundant polysaccharide. 2. Flexible to physical and chemical modifications. 3. Active sites of hydroxyl and primary amino functional groups. 4. pH responsiveness and stability in acidic environments. 5. Inherent biocompatibility.	1. High humidity sensitivity. 2. Cationic nature in low pH, causing low performance for cations adsorption.	(Haghighi et al., 2020; Yu et al., 2016)
Alginate/Sodium alginate	1. Water soluble. 2. Biocompatible and moderate gelation conditions. 3. Binds with multivalent cations.	1. High rigidity and fragile. 2. Low in elasticity and mechanical properties. 3. Lacks primary amino groups.	(Saket et al., 2022)
Cellulose	1. First most abundant polysaccharide. 2. Facile to chemical modifications. 3. Good mechanical properties 4. Safely disposable	1. Lacks primary amino groups.	(Saket et al., 2022)
Gelatin	1. Water-soluble polymer 2. Amphoteric nature 3. Active sites for electrostatic interactions of hydroxyl, carboxyl and amino functional groups.	1. Low reported adsorption capacities. 2. Difficulty of adsorbate ions separation or regeneration. 3. Poor mechanical stability.	(Jamwal et al., 2020; Saket et al., 2022)
Agarose	1. Hydrophobic and biocompatible. 2. Nontoxic and biodegradable in nature. 3. Great swelling capacity. 4. Chemically stable and neutrally charged. 5. Requires mild conditions of gelation.	1. Lack of amino functional groups 2. Relatively low stability in acidic environments. 3. Slow degradation rates.	(Saket et al., 2022; Zamora-Mora et al., 2014; Zhang et al., 2012)

**Table A3:** Recent Applications of Modified CS by Cross-linking for the Removal of Adsorbates from Aqueous Solutions.

Cross linker	Adsorbate	Chitosan Modifications	Refs
Diepoxyoctane	Cr(VI)	1. Increase in chitosan functional groups got involved in cross-linking reactions with increasing the cross-linkers concentration. 2. Increase in the adsorbate uptake and performance with the increase of the cross-linking temperature and time to a certain limit to avoid occupying adsorption sites.	(Vakili et al., 2018)
Penta sodium tripolyphosphate	Dye	1. Cross-linker agent accelerated the sorption process.	(Filipkowska et al., 2016)
Glutaraldehyde	Various pollutants (proteins, fluoride, dyes, etc.).	1. Cross-linked chitosan beads have shown a decrease in crystallinity. 2. Cross-linked beads have shown less regular structure, due to the breakage of intra-molecular and inter-molecular hydrogen bonds of native beads. 3. Smoother surfaces with more porous cross-sectioned microspheres.	(Amari et al., 2021; Gonçalves et al., 2005)
	Cd(II) ion	1. An increase in the cross-linked polymer space among its chains and surface area eases binding to the active sites.	(Mallakpour and Khadem, 2019)
Epichlorohydrin	N/A	1. Cross-linked microspheres have harder, more smooth, and less porous surfaces. 2. Matrix compaction was observed due to cross-linking.	(Gonçalves et al., 2005)
	Cr (VI)	1. Non-cross-linked chitosan beads show more adsorption performance stability in different pH compared to cross-linked chitosan beads. 2. Cross-linked beads improved the adsorption capacity of adsorbate in contact time of 30 min and pH = 3 compared to native chitosan beads.	(Hastuti et al., September 2015)

**Table A4:** Recent Applications of Modified CS Adsorbents via Grafting for the Removal of Contaminants from Aqueous Solutions.

Nanoparticle	Adsorbate	Chitosan Modifications	References
TiO_2_	Anionic Dyes N/A	1) Chemically stable, high surface area, nontoxic, enhanced adsorption performance. 2) Monolayer adsorption pattern and homogeneous distribution of the active sites. 1) Structurally defined and standalone nature of pores (not interconnected). 2) Smaller pores than native chitosan adsorbents but numerous and large enough.	(Gajera et al., 2022; Jawad et al., 2020; Patiño-Ruiz et al., 2020)
ZnO	N/A Anionic Dyes	1) Enhanced tensile strength. 1) Zn nanoparticles could restrict the movability of the chitosan chains. 2) Smooth and tight surface that increases with ZnO content due to interfacial interaction between ZnO nanoparticles and chitosan. 3) More thermal stability compared to native CS beads. 4) CS/ZnO nanocomposites reported a decomposition temperature of 275 °C while native CS is 230 °C.	(Mostafa et al., 2020; Rahman et al., 2018; Yadollahi et al., 2016)
FeO	Cr(VI) anions Naphthalene	1) Fe–O functional groups involved in Cr(VI) anions adsorption through electrostatic interactions. 2) Homogeneous monolayer adsorption due to its fitting to Langmuir models. 1) Low cost, low toxicity, large surface area, and biodegradability. 2) Easy removal of adsorbent for further reuse. 3) Enhanced stability in acidic and oxidative mediums due to the immobility of FeO nanoparticles in the chitosan structure. 4) Suitable for the adsorption of adsorbates that are responsive to magnetic fields in which the FeO change the magnetization dynamics of the beads.	(Liu et al., 2022; Patiño-Ruiz et al., 2020)
SiO_2_	Anionic dye (AR73) N/A	1) Enhanced Adsorption capacity of the dye anions. 1) Improve the mechanical, interfacial, and thermal properties. 2) Increased surface area of the adsorbents especially with the smaller size SiO_2_ nanoparticles. 3) Improved surface porosity and adsorption capacity. 4) Most abundant components in the earth's crust. 5) Low toxicity, excellent chemical stability, scalable synthetic availability, and low cost. 6) Adjustable pore structure 7) Availability of silanol groups with the ease of modification.	(Hu et al., 2021) (Cho and Park, 2015; Rafigh and Heydarinasab, 2017; Sipaut et al., 2015) (Li et al., 2021)

**Table A5:** Bands Obtained in the Raman Spectra data, Their Corresponding Assigned Molecular Bonds, Reported Wavelength, Measured Wavelengths, and their Intensity.

Vibration Type and Assignment^a)^	Reported Range	Pure Chitosan (commercial)	Native CS Beads	IIC Beads	IIC-EPI Beads	IIC-SiO_2_-EPI beads	Used IIC beads	Intensity^b)^
ν (CH_3_) in R–CH_3_	2885-2860 (Larkin, 2017)	2885	2891	2883	2885	2885	2886	vs
ν (CH_2_)	2936-2915 (Larkin, 2017)	2919	2927	2931	2931	2926	2929	vs
ν (OH in water)	3400-3200 (Larkin, 2017)	3306	3311	3312	3309	3310	3308	m
ν (OH) in O=C–OH glucopyranose units	3200-2600 (Larkin, 2017)	3373	3371	3367	3371	3371	3373	vw
ν (C=O) in amide I	1695-1630 (Larkin, 2017)	1666	1666	1666	1666	1666	1666	vw
$\delta_{as}$ (CH_3_)	1470-1250 (Larkin, 2017)	1415	1417	1419	1415	1415	1415	m
τ (CH_2_)	1475-1445 (Larkin, 2017)	1462	1462	1466	1464	1459	1462	m
τ (NH_2_)	1608-1621 (Larkin, 2017)	1666	1666	1666	1666	1666	1666	vw
δ (–CH_2_–OH)	1300-1050 (Larkin, 2017)	1267	1267	–	1262	1267	1264	vw
ν/τ –C (=O)–NH–C	3300 (s), 3100(m) (Larkin, 2017)	3306	3311	3312	3309	3310	3308	m
$\nu_{s}$ (C–*O*–C)	1140-800 (Larkin, 2017)	1100	1108	1108	1108	1109	1113	s
ν (C–N)	∼1150,∼1045 (Larkin, 2017)	1150	1150	1150	1150	1150	1152	w
δ (C–H)	804-991 (Wiercigroch et al., 2017)	896	894	894	897	897	897	s
δ-β(CCC), δ -β(COC) glycosidic bond	361-376 (Wiercigroch et al., 2017)	366	364	366	363	364	364	vw
δ (C–H) in rings, glycosidic linkage (COC), and δ (CCC)	948-980 (Wiercigroch et al., 2017)	945	945	947	947	957	943	vw
ν (H–I)	2229.6 (Nakamoto, 2009)							

^a)^ Vibration assignment: stretching vibrational mode (ν), deformational (δ), bending (τ), and symmetrical (s) and antisymmetric modes (as). ^b)^ bond intensity: s = strong, m = medium, w = weak, v = very, br = broad.

**Table A6:** Constants of Non-linear Pseudo First and Second Order Models of I^−^ Ions Adsorption.

Adsorbent	pH	Q_e_^a)^ [mmol.g^−1^]		Pseudo-First-Order				Pseudo-Second-Order		
				Q_e_^b)^ [mmol.g^−1^]	k_1_ (min^−1^)	R^2^		Q_e_ (mmol.g^−1^)	k_2_ (g mmol^−1^. min^−1^)	R^2^
Native CS	7	0.1512		0.1472	1.0144 × 10^13^	0.8950		0.1533	4.9615	0.9056
IIC	7	0.2416		0.2492	0.1792	0.9426		0.2641	1.2334	0.9661
IIC-EPI	7	0.4196		0.4323	0.0758	0.945		0.4745	0.2438	0.9722
IIC-SiO_2_-EPI	7	0.5130		0.5146	0.2632	0.9883		0.5185	3.5999	0.9861
IIC-SiO_2_-EPI	5	0.6944		0.6702	0.1570	0.9835		0.6997	0.5397	0.9945

^a)^Experimental Data; ^b)^ Calculated data by the model.

**Table A7:** Rate Constants for The Intraparticle Diffusion Kinetic Model of the I^−^ Ions Adsorption

Adsorbent	pH	K _int_			C			R^2^		
		Stage I	Stage II	Stage III	Stage I	Stage II	Stage III	Stage I	Stage II	Stage III
**Native CS**	7	−0.0230	0.0003	0.0080	0.2254	0.1436	0.0519	0.5694	0.0057	0.8983
**IIC**	7	0.0111	−0.0037	0.0095	0.1778	0.2615	0.1439	0.8935	0.8099	0.9724
**IIC-EPI**	7	0.0879	0.0241	0.0015	−0.0354	0.2262	0.4361	0.8669	0.9000	0.7171
**IIC-SiO2-EPI**	7	0.0311	−0.0183	0.0082	0.3777	0.6388	0.4001	0.9594	0.9527	0.9996
**IIC-SiO2-EPI**	5	0.0170	0.0171	0.0055	0.5164	0.5532	0.6116	0.9733	0.6325	0.9420

**Table A8:** Constants of Non-Linear Langmuir and Freundlich Isotherms of I^−^ Ions Adsorption

Adsorbent	pH	Langmuir isotherm			Freundlich isotherm		
		Q_m_ (mmol/g)	K_L_ (L/mmol)	R^2^	K_F_ (L^1/n^·mmol^1−1/n^·mmol^−1^)	n	R^2^
Native CS	7	0.5750	0.6395	0.9526	0.2128	1.3781	0.9239
IIC	7	3.5581	0.1942	0.9600	0.5778	1.0625	0.9548
IIC-EPI	7	2.4593	0.3945	0.9674	0.7003	1.1611	0.9564
IIC-SiO_2_-EPI	7	4.2247	0.3198	0.9600	1.0599	1.1005	0.9537
IIC-SiO_2_-EPI	5	1.9443	2.7366	0.9668	1.7172	1.6135	0.9300

**Fig. A1:**
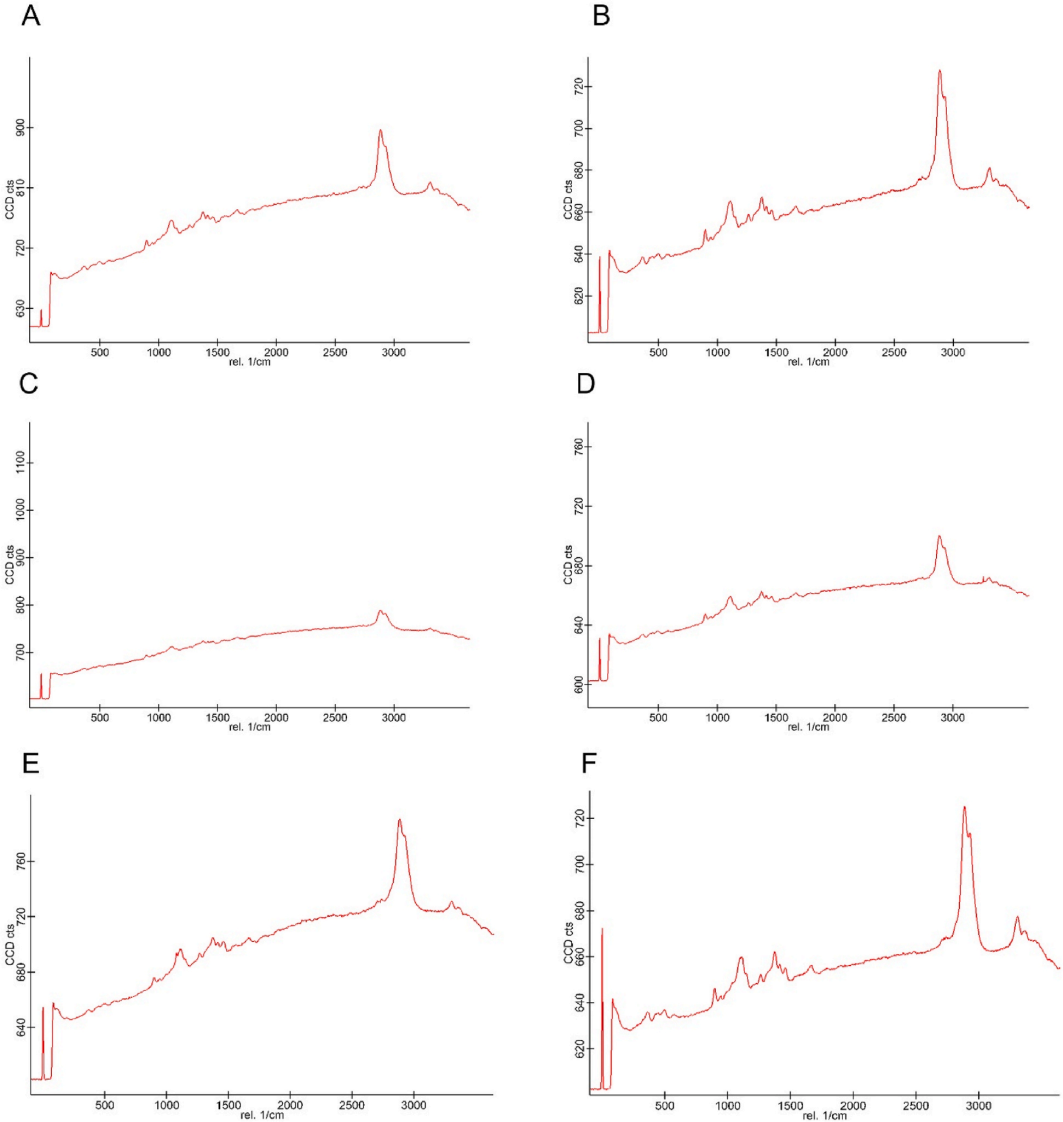
Raman spectroscopy raw data of: A) pure commercial chitosan, B) Native chitosan beads, C) I^−^-imprinted chitosan beads (IIC), D) I^−^-imprinted chitosan beads cross-linked with Epichlorohydrin (IIC-EPI), E) SiO_2_ grafted I^−^-imprinted chitosan beads cross-linked with Epichlorohydrin (IIC–SiO_2_-EPI), F) used I^−^-imprinted chitosan beads (IIC) (post adsorption), G) Template molecules (NaI). Silicon was used for calibration (narrow peak at the ∼0 cm ^−1^ wavelength).

## Appendix

Molecularly Imprinted Chitosan Beads for Highly Selective Adsorption of Iodide for Nuclear Wastewater Treatment.

Yassmin Handulle Ismail, Kean Wang, Maryam Al Shehhi, Ali Al Hammadi*

## References

[1] Corkhill C., Hyatt N. (2018).

[2] El-Shahat M., Abdelhamid A.E., Abdelhameed R. (2020). Capture of iodide from wastewater by effective adsorptive membrane synthesized from MIL-125-NH2 and cross-linked chitosan. Carbohydr. Polym..

[3] Xu S. (2015). Speciation of radiocesium and radioiodine in aerosols from Tsukuba after the Fukushima nuclear accident. Environ. Sci. Technol..

[4] Muramatsu Y., Ohmomo Y. (1986). Iodine-129 and iodine-127 in environmental samples collected from Tokaimura/Ibaraki, Japan. Sci. Total Environ..

[5] Li X. (2022). Magnetic chitosan microspheres: an efficient and recyclable adsorbent for the removal of iodide from simulated nuclear wastewater. Carbohydr. Polym..

[6] Eisenbud M. (1962). Iodine-131 Dose from Soviet Nuclear Tests: accumulation of iodine-131 in human thyroids was observed by in vivo procedures during the 1961 tests. Science.

[7] Efremenkov V.M. (1989). Radioactive waste management at nuclear power plants. IAEA Bull..

[8] Li D. (2018). Removal capacity and chemical speciation of groundwater iodide (I−) and iodate (IO3−) sequestered by organoclays and granular activated carbon. J. Environ. Radioact..

[9] Zhang H. (2011). Adsorption of iodide ions on a calcium alginate–silver chloride composite adsorbent. Colloids Surf. A Physicochem. Eng. Asp..

[10] Xia M. (2019). Removal of Hg (II) in aqueous solutions through physical and chemical adsorption principles. RSC Adv..

[11] Tesfay Reda A. (2021). Bismuth-based materials for iodine capture and storage: a review. J. Environ. Chem. Eng..

[12] Osmanlioglu A.E. (2018). Decontamination of radioactive wastewater by two-staged chemical precipitation. Nucl. Eng. Technol..

[13] Lehto J., Harjula R. (1999). Selective separation of radionuclides from nuclear waste solutions with inorganic ion exchangers. Radiochim. Acta.

[14] Azlan K., Saime W.N.W., Liew L. (2009). Chitosan and chemically modified chitosan beads for acid dyes sorption. J. Environ. Sci..

[15] Wang J., Zhuang S. (2017). Removal of various pollutants from water and wastewater by modified chitosan adsorbents. Crit. Rev. Environ. Sci..

[16] Martínez J.P., Falomir M.P., Gozalbo D. (2009).

[17] Zia Q. (2019). A Review on Chitosan for the removal of heavy metals ions. JFBI.

[18] Akpan E.I., Gopi S., Thomas S., Pius A. (2020). Handbook of Chitin and Chitosan.

[19] Basu P., Basu P. (2018). Biomass Gasification, Pyrolysis and Torrefaction.

[20] Chen H., Chen H. (2015). Lignocellulose Biorefinery Engineering.

[21] Rojas J., Madrigal J., Ortiz J. (2015). Effect of acid hydrolysis on tableting properties of chitin obtained from shrimp heads. Trop. J. Pharmaceut. Res..

[22] Elnashar M. (2011).

[23] Yu J. (2016). Selective adsorption and reusability behavior for Pb2+ and Cd2+ on chitosan/poly (ethylene glycol)/poly (acrylic acid) adsorbent prepared by glow-discharge electrolysis plasma. Colloid Polym. Sci..

[24] Haghighi H. (2020). Recent advances on chitosan-based films for sustainable food packaging applications. Food Packag. Shelf Life.

[25] Saket P. (2022). Microalgae and bio-polymeric adsorbents: an integrative approach giving new directions to wastewater treatment. Int. J. Phytoremediation.

[26] Jamwal H.S. (2020). Gelatin-based mesoporous hybrid materials for Hg2+ ions removal from aqueous solutions. Sep. Purif. Technol..

[27] Zamora-Mora V. (2014). Chitosan/agarose hydrogels: cooperative properties and microfluidic preparation. Carbohydr. Polym..

[28] Zhang L.-M. (2012). Synthesis and characterization of a degradable composite agarose/HA hydrogel. Carbohydr. Polym..

[29] Nunthanid J. (2001). Physical properties and molecular behavior of chitosan films. Drug Dev. Ind. Pharm..

[30] Justin R., Chen B. (2014). Characterisation and drug release performance of biodegradable chitosan–graphene oxide nanocomposites. Carbohydr. Polym..

[31] Fu G. (2007). Bovine serum albumin-imprinted polymer gels prepared by graft copolymerization of acrylamide on chitosan. React. Funct. Polym..

[32] Guo T.-Y. (2005). Chemically modified chitosan beads as matrices for adsorptive separation of proteins by molecularly imprinted polymer. Carbohydr. Polym..

[33] Sellergren B. (2000).

[34] Zhu S., Hamielec A., Matyjaszewski K., Möller M. (2012). Polymer Science: A Comprehensive Reference.

[35] Roh I.J., Kwon I.-C. (2002). Fabrication of a pure porous chitosan bead matrix: influences of phase separation on the microstructure. J. Biomater. Sci. Polym. Ed..

[36] Zare S., Kargari A., Gude V.G. (2018). Emerging Technologies for Sustainable Desalination Handbook.

[37] Zhu H.-Y. (2010). A novel magnetically separable γ-Fe2O3/crosslinked chitosan adsorbent: preparation, characterization and adsorption application for removal of hazardous azo dye. J. Hazard Mater..

[38] Vakili M. (2018). Novel crosslinked chitosan for enhanced adsorption of hexavalent chromium in acidic solution. Chem. Eng. J..

[39] Filipkowska U. (2016). Impact of chitosan cross-linking on RB 5 dye adsorption efficiency. Prog. Chem..

[40] Amari A. (2021). Multifunctional crosslinked chitosan/nitrogen-doped graphene quantum dot for wastewater treatment. Ain Shams Eng. J..

[41] Gonçalves V.L. (2005). Effect of crosslinking agents on chitosan microspheres in controlled release of diclofenac sodium. Polímeros.

[42] Hastuti B., Masykur A., Hadi S. (September, 2015). IOP Conf. Ser. Mater. Sci. Eng.

[43] Farias P. (2015). Natural and cross-linked chitosan spheres as adsorbents for diesel oil removal. Adsorpt. Sci. Technol..

[44] Rafigh S.M., Heydarinasab A. (2017). Mesoporous chitosan–SiO2 nanoparticles: synthesis, characterization, and CO2 adsorption capacity. ACS Sustain. Chem. Eng..

[45] Sipaut C.S. (2015). The effect of surface modification of silica nanoparticles on the morphological and mechanical properties of bismaleimide/diamine matrices. Adv. Polym. Technol..

[46] Cho A.-R., Park S.-Y. (2015). Synthesis of titania-and silica-polymer hybrid materials and their application as refractive index-matched layers in touch screens. Opt. Mater. Express.

[47] Mallakpour S., Khadem E. (2019). Linear and nonlinear behavior of crosslinked chitosan/N-doped graphene quantum dot nanocomposite films in cadmium cation uptake. Sci. Total Environ..

[48] Patino-Ruiz D.A. (2020). Ionic cross-linking fabrication of chitosan-based beads modified with FeO and TiO2 nanoparticles: adsorption mechanism toward naphthalene removal in seawater from cartagena bay area. ACS Omega.

[49] Gajera R. (2022). Adsorption of cationic and anionic dyes on photocatalytic flyash/TiO2 modified chitosan biopolymer composite. JWPE.

[50] Jawad A.H., Mubarak N.S.A., Abdulhameed A.S. (2020). Hybrid crosslinked chitosan-epichlorohydrin/TiO2 nanocomposite for reactive red 120 dye adsorption: kinetic, isotherm, thermodynamic, and mechanism study. J. Polym. Environ..

[51] Rahman P.M. (2018). Chitosan/nano ZnO composite films: enhanced mechanical, antimicrobial and dielectric properties. Arab. J. Chem..

[52] Yadollahi M. (2016). Facile synthesis of chitosan/ZnO bio-nanocomposite hydrogel beads as drug delivery systems. Int. J. Biol. Macromol..

[53] Mostafa M.H. (2020). Microwave-Assisted preparation of Chitosan/ZnO nanocomposite and its application in dye removal. Mater. Chem. Phys..

[54] Liu Y. (2022). Removal of Cr (VI) from wastewater using graphene oxide chitosan microspheres modified with α–FeO (OH). Materials.

[55] Hu X. (2021). Preparation of Acid Red73 adsorbed on chitosan-modified sepiolite with SiO2 coating as a highly stable hybrid pigment. Dyes Pigments.

[56] Li H. (2021). Functionalized silica nanoparticles: classification, synthetic approaches and recent advances in adsorption applications. Nanoscale.

[57] Jørgensen S.E. (1979). Industrial Waste Water Management.

[58] Borges Silverio L., Lamas W.d.Q. (2011). An analysis of development and research on spent nuclear fuel reprocessing. Energy Pol..

[59] Giannakoudakis D.A. (2021). Enhanced uranium removal from acidic wastewater by phosphonate-functionalized ordered mesoporous silica: surface chemistry matters the most. J. Hazard Mater..

[60] Jeon C., Park K.H. (2005). Adsorption and desorption characteristics of mercury (II) ions using aminated chitosan bead. Water Res..

[61] Su H. (2020). Removal of high concentrations of NO3− from nuclear industrial wastewater by using a fixed-bed bioreactor. J. Radioanal. Nucl. Chem..

[62] Dong F. (2021). Highly efficient uptake of TcO4–by imidazolium-functionalized wood sawdust. ACS Omega.

[63] Halevi O. (2020). Nuclear wastewater decontamination by 3D-Printed hierarchical zeolite monoliths. RSC Adv..

[64] Yang J. (2022). Enhanced removal of radioactive iodine anions from wastewater using modified bentonite: experimental and theoretical study. Chemosphere.

[65] Xu L. (2021). A pillared double-wall metal-organic framework adsorption membrane for the efficient removal of iodine from solution. Sep. Purif. Technol..

[66] Dong C. (2021). Molecularly imprinted polymers by the surface imprinting technique. Eur. Polym. J..

[67] BelBruno J.J. (2018). Molecularly imprinted polymers. Chem. Rev..

[68] Chen L., Xu S., Li J. (2011). Recent advances in molecular imprinting technology: current status, challenges and highlighted applications. Chem. Soc. Rev..

[69] Zhang M., Helleur R., Zhang Y. (2015). Ion-imprinted chitosan gel beads for selective adsorption of Ag+ from aqueous solutions. Carbohydr. Polym..

[70] Shekhawat A. (2016). Assimilation of chitin with tin for defluoridation of water. RSC Adv..

[71] Zhang W. (2019). Cross-linked chitosan microspheres: an efficient and eco-friendly adsorbent for iodide removal from waste water. Carbohydr. Polym..

[72] Alamrani N.A. (2023). Developing thiosemicarbazide-modified/ion-imprinted chitosan for selective cadmium ion biosorption. Mater. Today Chem..

[73] Alnawmasi J.S. (2023). Construction of amino-thiol functionalized ion-imprinted chitosan for lead (II) ion removal. Carbohydr. Polym..

[74] Hajri A.K. (2022). Designing of modified ion-imprinted chitosan particles for selective removal of mercury (II) ions. Carbohydr. Polym..

[75] Zhou G. (2022). Ion imprinted polymer layer modified magnetic nanocomposites for selective recycling of aqueous Ni(II). J. Clean. Prod..

[76] Beltran A. (2010). Molecularly-imprinted polymers: useful sorbents for selective extractions. TrAC, Trends Anal. Chem..

[77] Van Deventer J., Van der Merwe P. (1993). The effect of temperature on the desorption of gold cyanide from activated carbon. Thermochim. Acta.

[78] Babakhani A., Sartaj M. (2022). Synthesis, characterization, and performance evaluation of ion-imprinted crosslinked chitosan (with sodium tripolyphosphate) for cadmium biosorption. J. Environ. Chem. Eng..

[79] Patel H. (2021). Review on solvent desorption study from exhausted adsorbent. J. Saudi Chem. Soc..

[80] Melhem G. (2023). An analysis of α-epichlorohydrin-water runaways. Process Saf. Environ. Protect..

[81] Pérez-Moreno A. (2021). Effect of washing treatment on the textural properties and bioactivity of silica/chitosan/TCP xerogels for bone regeneration. Int. J. Mol. Sci..

[82] Ardila N. (2017). Antibacterial activity of neat chitosan powder and flakes. Molecules.

[83] Julkapli N.M., Ahmad Z., Akil H.M. (2010). AIP Conference Proceedings.

[84] Dey S.C. (2016). Preparation, characterization and performance evaluation of chitosan as an adsorbent for remazol red. Int. J. Latest res. Eng. Technol.

[85] Aziz S.B. (2017). Polymer blending as a novel approach for tuning the SPR peaks of silver nanoparticles. Polymers.

[86] Crawford C.B., Quinn B. (2016).

[87] Igberase E., Ofomaja A., Osifo P. (2019). Enhanced heavy metal ions adsorption by 4-aminobenzoic acid grafted on chitosan/epichlorohydrin composite: kinetics, isotherms, thermodynamics and desorption studies. Int. J. Biol. Macromol..

[88] Abdel Aziz Mahmoud O.O. (2014). FTIR spectroscopy of natural bio-polymers blends. Middle East J. Appl. Sci..

[89] Silva S.M., Theophile T. (2012). Infrared Spectroscopy - Materials Science, Engineering and Technology.

[90] Tirtom V.N. (2012). Comparative adsorption of Ni (II) and Cd (II) ions on epichlorohydrin crosslinked chitosan–clay composite beads in aqueous solution. Chem. Eng. J..

[91] Danalıoğlu S.T. (2018). Chitosan grafted SiO 2–Fe 3 O 4 nanoparticles for removal of antibiotics from water. Environ. Sci. Pollut. Res..

[92] Larkin P. (2017).

[93] Abiddin J.F.Z., Ahmad A.H. (2015). Fourier transform infrared spectroscopy and electrical characterization of methylcellulose based solid polymer electrolyte doped with sodium iodide. Jurnal Teknologi.

[94] Wiercigroch E. (2017). Raman and infrared spectroscopy of carbohydrates: a review. Spectrochim. Acta, Part A.

[95] Nakamoto K. (2009).

[96] Kumar S., Dutta P., Koh J. (2011). A physico-chemical and biological study of novel chitosan–chloroquinoline derivative for biomedical applications. Int. J. Biol. Macromol..

[97] Chandra S., Naiker M., Cozzolino D. (2022). Comprehensive Analytical Chemistry.

[98] Kireev S.V., Shnyrev S.L. (2015). Study of molecular iodine, iodate ions, iodide ions, and triiodide ions solutions absorption in the UV and visible light spectral bands. Laser Phys..

[99] Chen A.-H. (2008). Comparative adsorption of Cu(II), Zn(II), and Pb(II) ions in aqueous solution on the crosslinked chitosan with epichlorohydrin. J. Hazard Mater..

[100] do Amaral Sobral P.J. (2022). Rheological and viscoelastic properties of chitosan solutions prepared with different chitosan or acetic acid concentrations. Foods.

[101] Ren L. (2019). Preparation and characterization of porous chitosan microspheres and adsorption performance for hexavalent chromium. Int. J. Biol. Macromol..

[102] Sakkayawong N., Thiravetyan P., Nakbanpote W. (2005). Adsorption mechanism of synthetic reactive dye wastewater by chitosan. J. Colloid Interface Sci..

[103] Abbasi M. (2017). Synthesis and characterization of magnetic nanocomposite of chitosan/SiO2/carbon nanotubes and its application for dyes removal. J. Clean. Prod..

[104] Zhu L., Bratlie K.M. (2018). pH sensitive methacrylated chitosan hydrogels with tunable physical and chemical properties. Biochem. Eng. J..

[105] Van Gheluwe L. (2021). Polymer-based smart drug delivery systems for skin application and demonstration of stimuli-responsiveness. Polymers.

[106] Zhu H.-Y. (2010). A novel magnetically separable γ-Fe2O3/crosslinked chitosan adsorbent: preparation, characterization and adsorption application for removal of hazardous azo dye. J. Hazard Mater..

[107] Yagub M.T., Sen T.K., Ang M. (2014). Removal of cationic dye methylene blue (MB) from aqueous solution by ground raw and base modified pine cone powder. Environ. Earth Sci..

[108] Song X. (2012). Low-cost carbon nanospheres for efficient removal of organic dyes from aqueous solutions. Ind. Eng. Chem. Res..

[109] Sun S., Wang A. (2006). Adsorption kinetics of Cu (II) ions using N, O-carboxymethyl-chitosan. J. Hazard Mater..

[110] Bhanvase B.A. (2021).

[111] Sahoo T.R., Prelot B. (2020). Nanomaterials for the Detection and Removal of Wastewater Pollutants.

[112] Kalia S. (2021).

[113] Kajjumba G.W. (2018). Modelling of adsorption kinetic processes—errors, theory and application. Adv. Sorp. Proc. App..

[114] Plazinski W., Dziuba J., Rudzinski W. (2013). Modeling of sorption kinetics: the pseudo-second order equation and the sorbate intraparticle diffusivity. Adsorption.

[115] Tan K.L., Hameed B.H. (2017). Insight into the adsorption kinetics models for the removal of contaminants from aqueous solutions. J. Taiwan Inst. Chem. Eng..

[116] Althomali R.H. (2023). An investigation on the adsorption and removal performance of a carboxymethylcellulose-based 4-aminophenazone@ MWCNT nanocomposite against crystal violet and brilliant green dyes. RSC Adv..

[117] Campos N.F. (2018). Removal of naphthenic acids using activated charcoal: kinetic and equilibrium studies. Adsorpt. Sci. Technol..

[118] Tsibranska I., Hristova E. (2011). Comparison of different kinetic models for adsorption of heavy metals onto activated carbon from apricot stones. Bulg. Chem. Commun..

[119] Foroughirad S. (2021). Effect of porogenic solvent in synthesis of mesoporous and microporous molecularly imprinted polymer based on magnetic halloysite nanotubes. Mater. Today Commun..

[120] Ahmed M.A., Abdelbar N.M., Mohamed A.A. (2018). Molecular imprinted chitosan-TiO2 nanocomposite for the selective removal of Rose Bengal from wastewater. Int. J. Biol. Macromol..

[121] Osmari T.A. (2013). Statistical analysis of linear and non-linear regression for the estimation of adsorption isotherm parameters. Adsorpt. Sci. Technol..

[122] Chung H.-K. (2015). Application of Langmuir and Freundlich isotherms to predict adsorbate removal efficiency or required amount of adsorbent. J. Ind. Eng. Chem..

[123] Rudzinski W., Plazinski W. (2007). Studies of the kinetics of solute adsorption at solid/solution interfaces: on the possibility of distinguishing between the diffusional and the surface reaction kinetic models by studying the pseudo-first-order kinetics. JPC C.

[124] Mu W. (2016). Safe disposal of radioactive iodide ions from solutions by Ag 2 O grafted sodium niobate nanofibers. Dalton Trans..

[125] Mao P. (2016). Enhanced uptake of iodide on Ag@Cu2O nanoparticles. Chemosphere.

[126] Li Q. (2019). Hydrophobic-modified montmorillonite coating onto crosslinked chitosan as the core-shell micro-sorbent for iodide adsorptive removal via Pickering emulsion polymerization. Int. J. Biol. Macromol..

[127] Choung S. (2013). Uptake mechanism for iodine species to black carbon. Environ. Sci. Technol..

